# Locally Enhanced Flow and Electric Fields Through a Tip Effect for Efficient Flow-Electrode Capacitive Deionization

**DOI:** 10.1007/s40820-024-01531-0

**Published:** 2024-09-27

**Authors:** Ziquan Wang, Xiangfeng Chen, Yuan Zhang, Jie Ma, Zhiqun Lin, Amor Abdelkader, Maria-Magdalena Titirici, Libo Deng

**Affiliations:** 1 College of Chemistry and Environmental Engineering, Shenzhen University, Shenzhen, 518060 People’s Republic of China; 2 Research Center for Environmental Functional Materials, College of Environmental Science and Engineering, Tongji University, 1239 Siping Road, Shanghai, 200092 People’s Republic of China; 3 Department of Chemical and Biomolecular Engineering, National University of Singapore, Singapore, 117585 Singapore; 4 Department of Engineering, Faculty of Science and Technology, Bournemouth University, Talbot Campus, Fern Barrow, Poole, England BH12 5BB UK; 5 Institut de Chimie de Nice, Université Côte d’Azur, UMR CNRS 7272, 28 Av. Valrose, 06108 Nice, France; 6 Department of Chemical Engineering, Imperial College London, South Kensington Campus, Exhibition Rd, London, SW7 2AZ UK

**Keywords:** Flow-electrode, Capacitive deionization, Current collector, Tip effect, Desalination

## Abstract

Low-electrode capacitive deionization (FCDI) is an emerging desalination technology with great potential for removal and/or recycling ions from a range of waters. However, it still suffers from inefficient charge transfer and ion transport kinetics due to weak turbulence and low electric intensity in flow electrodes, both restricted by the current collectors. Herein, a new tip-array current collector (designated as T-CC) was developed to replace the conventional planar current collectors, which intensifies both the charge transfer and ion transport significantly. The effects of tip arrays on flow and electric fields were studied by both computational simulations and electrochemical impedance spectroscopy, which revealed the reduction of ion transport barrier, charge transport barrier and internal resistance. With the voltage increased from 1.0 to 1.5 and 2.0 V, the T-CC-based FCDI system (T-FCDI) exhibited average salt removal rates (ASRR) of 0.18, 0.50, and 0.89 μmol cm^−2^ min^−1^, respectively, which are 1.82, 2.65, and 2.48 folds higher than that of the conventional serpentine current collectors, and 1.48, 1.67, and 1.49 folds higher than that of the planar current collectors. Meanwhile, with the solid content in flow electrodes increased from 1 to 5 wt%, the ASRR for T-FCDI increased from 0.29 to 0.50 μmol cm^−2^ min^−1^, which are 1.70 and 1.67 folds higher than that of the planar current collectors. Additionally, a salt removal efficiency of 99.89% was achieved with T-FCDI and the charge efficiency remained above 95% after 24 h of operation, thus showing its superior long-term stability.

**Supplementary Information:**

The online version contains supplementary material available at 10.1007/s40820-024-01531-0.

## Highlights

- Steel tip arrays were used as current collectors to replace planar conductors.
- Optimal flow and electric fields reduced barriers for electron and ion transport.
- Desalination performance of flow-electrode capacitive deionization is enhanced by the tip-array current collectors.

**Supplementary Information:**

The online version contains supplementary material available at 10.1007/s40820-024-01531-0.

## Introduction

In recent years, freshwater scarcity has been exacerbated by the rapid increase in global population and worsening water pollution [1–3]. Conventional desalination technologies (e.g., reverse osmosis, electrodialysis and thermal desalination) are energy-intensive and suffer from membrane fouling, low water recovery rates and high capital costs [4, 5]. Therefore, new desalination technologies with both high energy efficiency and environmental friendliness are highly desirable [6, 7].

Capacitive deionization (CDI) is emerging as a promising desalination technology that captures and stores ions in the porous carbon electrodes, driven by the electric field [8, 9]. This technology shows advantages over traditional ones in low energy consumption, zero emission of secondary pollution, mild operating conditions, low investment costs, etc. [10–12]. Particularly, by replacing the conventional planar electrodes with flowing electrodes in which active materials are dispersed in aqueous, the ion transport can be significantly accelerated, thus increasing the salt removal rate. Since the debut of the so-called flow-electrode capacitive deionization (FCDI) technology, it has been extensively investigated for its promising potential applications in desalination [13], water softening [14], wastewater treatment [15] and resource recovery [16], etc. Furthermore, when operated with a short-circuited closed-cycle (SCC) mode, the electrodes can be continuously regenerated by neutralizing the charges in an external electrode reservoir, which allows continuous operation and thereby overcoming the capacity limitations of the conventional CDI systems [17].

The current collector is essential to the FCDI system, which conducts the electrons between the flow electrode and the external power source, and its structure determines the charge transfer process, flow field and electric field distribution in the FCDI system [18]. Traditional current collectors are mostly made of graphite in a form of two-dimensional (2D), usually coupled with serpentine flow channels, which exhibit poor contact with the flow electrode and, thus, sluggish kinetics for charge transfer [19]. Additionally, the traditional current collector generates a weak electric field at low potentials, leading to slow ion migration kinetics [20]. Therefore, optimizing the traditional 2D planar design of the current collectors is crucial for improving the desalination performance of FCDI systems.

Extensive efforts have been devoted to optimizing current collectors, aiming to enhance the charge transfer and optimize the flow field. Currently, two strategies are frequently used to achieve these goals: structural optimization and surface modification. Specifically, one effective way is to turn the 2D structure of the current collectors into three-dimensional (3D) structure (e.g., Ti mesh/foam [19, 21, 22] and Ni foam [23]). The 3D structure could reduce the charge transfer distance and increase the charge transfer area, thus lowering the system’s internal resistance and leading to a higher salt removal rate (e.g.*,* 1.76-fold increase was achieved by using the Ti mesh [19]), charge efficiency and lower energy consumption. However, the efficiency of transport kinetics is still unsatisfying due to the limited curvature of the 3D structure that restricts the electrical field intensity, as well as the high ion/charge transport resistances. Another approach is to change the architecture of the current collectors, such as Archimedes spiral [24], mosquito-repellent incense-shaped [25], double Fermat’s spiral [26] flow channel or hexagonal honeycomb-shaped flow channel [27]. The rationally designed flow channels increased the velocity of the flow electrode and constructed the charge percolation network, which is responsible for the improved desalination performance (e.g., a 1.83-fold increase was achieved by using the Archimedes spiral flow channel [24]). Besides, surface coating was also proved to be effective. By coating the graphite current collectors with polyaniline [28], the conductivity of the current collectors increased 3.6 times, leading to an enhanced charge transfer efficiency. The coating material also changes the surface polarity of the current collectors and then reduces flow electrode sedimentation. However, the friction between flow electrode and current collector poses challenges in maintaining the stability of surface coatings, potentially leading to detachment or chemical reactions that alter their properties and result in performance degradation.

It has been well documented that the surface electric field intensity is proportional to the curvature of the conductor according to Gauss’s law. This effect is also known as the local electric field enhancement (LEFE) [29], which has been widely used in the field of electrochemical sterilization [30] and electrocatalysis [31]. It is thus expected that a tip-shaped current collector can also enhance the desalination performance for FCDI, considering that the tip-shaped electrode can generate higher local electric field and thus accelerate charge transfer and ion transport. However, there has been no report on applying LEFE strategy to the FCDI system so far, and the underlying mechanism requires explorations.

Herein, a novel current collector with tip-array structure is developed to enhance the FCDI. This was conducted by first investigating the effect of the current collector on the flow field and electric field by computational simulations. The kinetic difference of the FCDI systems equipped with different current collectors was then revealed by electrochemical impedance spectroscopy with distribution of relaxation times (EIS-DRT) analysis. Furthermore, the desalination performances of the FCDI using three types of current collectors (serpentine shape, planar shape, and tip shape) were evaluated. It was found that the FCDI system equipped with tip-array current collectors showed significant enhancement over the other systems.

## Experimental

### Chemicals and Materials

Sodium chloride (NaCl, 99.5%) and Fluorescein sodium (AR) were purchased from Shanghai Aladdin Biochemical Technology Co., Ltd. Activated carbon (YP-50F) was purchased from Kuraray Co., Ltd. Conductive carbon black (Vulcan XC-72R) was purchased from Cabot Corp. The properties of the activated carbon are listed in Table S1. Deionized water was lab-made by an ultrapure water system (YOUPU UPHC-I-90 T, Chengdu, China). All chemicals were used as received without further purification. The cation exchange membrane (CMX) and the anion exchange membrane (AMX) were purchased from ASTOM Co., Ltd. The properties of the ion exchange membranes (IEMs) are listed in Table S2.

### Characterization Techniques

The morphology and microstructure of the current collectors were characterized by field emission scanning electron microscopy (FE-SEM, JSM-7800F&TEAM Octane Plus, JEOL Co., Ltd.). The chemical composition of the current collectors was investigated by the energy-dispersive spectrometer (EDS, EDAX Inc., USA). X-ray diffraction (XRD) measurements were carried out on a Bruker/D8 Advance diffractometer (Bruker Technology Co., Ltd.) equipped with a CuKα radiation source. The contact angle was measured by drop shape analysis (Optical contact angle measuring instrument, SINDIN & SDC-100).

### Fabrication of the Current Collectors

Three types of current collectors were used in this study: current collector without serpentine flow channels (planar current collector, denoted as P-CC), current collector with serpentine flow channels (serpentine current collector, denoted as S-CC) and the current collector with tip arrays (tip current collector, denoted as T-CC). Detailed structure can be found in the supplementary computer-aided design files. The geometric parameters of three kinds of current collectors can be found in Table S3.

T-CCs and P-CCs were fabricated by the selective laser melting (SLM) method using a metal 3D printer (iSLM160, ZRapid Technologies Co., Ltd.). The computer-aided design files of the products, which can be found in the Online Resource (ESM_1-3.dwg), were imported to the 3D printer, and a proper set of process parameters were used to fabricate the products. The gas-atomized 316 L powder used in this work was spherical and ranged from 15 to 53 μm. The chemical composition and detailed physical characteristics of the current collectors can be found in Table S4. S-CCs were made of graphite, which were directly bought from Changsha Sipulin New Energy Technology Co., Ltd., China (its structure can be found in Fig. S1).

### Flow-Electrode Capacitive Deionization System Setup

The structure of the FCDI cells is illustrated in Fig. S2. It consists of a flowing anode, a saltwater chamber, and a flowing cathode. These chambers are separated by the anion exchange membrane (AEM) and cation exchange membrane (CEM). The size of the FCDI cell was 9 cm × 9 cm. The thickness of the current collectors, acrylic sheet (as saltwater chamber), and rubber gasket (prevent liquid leaking) were 7, 5, and 0.5 mm, respectively. The contact area between the flow electrode and the IEM was 4 cm × 4 cm. All components were held together by two acrylic plates and fixed by eight M3 bolts.

### In Situ Flow Field Measurements

In situ measurements were performed to validate the enhanced effect of tip structure on flow field. 1 wt% fluorescein sodium aqueous solution was used as feed solution and the liquid distribution was tracked using a digital camera. The inlet flow rate was set as 15 mL min^−1^. Under irradiation by a portable ultraviolet analyzer (365 nm, WFH-204BS), the fluorescein sodium emitted green fluorescence. By recording the distribution of fluorescence at different time intervals, we could compare the flow fields equipped with different current collectors (P-CC and T-CC).

### Electrochemical Measurements and Analysis Methods

Electrochemical impedance spectroscopy (EIS) measurements were conducted using an electrochemical workstation (CHI 760E, Shanghai Chenhua Instrument Co., Ltd., China) in the real FCDI systems (two electrodes configuration). All measurements were conducted in the frequency range from 10^5^ to 10^–2^ Hz at the steady-state open circuit potential (OCP) by applying a small sinusoidal signal with an amplitude of 5 mV, which were performed after holding the system at their OCP to achieve steady-state condition (i.e.*,* the OCP changing magnitude is less than 1 mV in 30 min). EIS data were validated by examining the residuals between the measured data and Kramers–Kronig (KK)-transformed results. The relative residuals of all EIS data in this work were less than 1% (all data satisfy the KK relationship), indicating that the FCDI systems under investigation were linear, stable, and causal [32].

The validated EIS data were converted to distribution of relaxation times (DRT) plots using DRT tools via MATLAB R2023a [33]. Gaussian method was used for data discretization, and the Tikhonov regularization parameter was set at 0.001. The radial basis function (RBF) with a full width at half maximum (FWHM) of 0.5 was used to fit the EIS data appropriately. See Supplementary Note S3.1 for details.

### Fabrication of the Flow Electrode

A certain amount of YP-50F and carbon black (with a mass ratio of 9:1) were added into the salt solution (3.5 g L^−1^ NaCl aqueous solution) and stirred at 550 rpm for 24 h by a magnetic stirrer (Shanghai Yuezhong Instrument Co., Ltd., China) to form 100 g of well-dispersed flow electrode. The mass of the YP-50F and carbon black was adjusted to fabricate the flow electrode with different solid contents.

### Desalination Experiments

The FCDI systems in this study were operated in SCC and batch mode. The volumes of the flow electrode and saltwater were 100 and 50 mL, respectively. Both were cycled at a flow rate of 30 mL min^−1^ by the constant flow pumps (BT100-2 J, Longer Precision Pump Co., Ltd., China). Additionally, an electrochemical workstation was used to apply a constant voltage to the system while the transient current was recorded with a sampling interval of 0.1 s. The conductivity of the saltwater was monitored in real-time by a conductivity meter (Jenway 4520, Cole-Parmer Instrument Co., Ltd., USA) with a sampling interval of 1 min. The salt concentration was converted by the calibration curve (Fig. S3). All experiments were conducted for 3600 s except for the long-term desalination tests. The long-term desalination experiments and stability tests were conducted at 1.5 V with a 5 wt% solid content of the flow electrode for 24 h and 7 days (12 h as a cycle), respectively. Electrodialysis (ED) tests were conducted in the same FCDI cell with a 0 wt% solid content of the flow electrode, and the other configurations remained unchanged.

### Evaluation of the Desalination Performance

The average salt removal rate (ASRR, μmol cm^−2^ min^−1^), energy-normalized removed salt (ENRS, μmol J^−1^), charge efficiency (CE, %), salt removal efficiency (SRE, %), and productivity (L m^−2^ h^−1^) were used to evaluate the desalination performance of the FCDI systems. The metrics mentioned above were calculated as follows:

1 $$ \begin{array}{*{20}c} {{\text{ASRR}} = \frac{{\left( {C_{0} - C_{{\text{t}}} } \right) \times V_{{\text{s}}} }}{M \times A \times t}} \\ \end{array} $$

2 $$ \begin{array}{*{20}c} {{\text{ENRS}} = \frac{{\left( {C_{0} - C_{{\text{t}}} } \right) \times V_{{\text{s}}} }}{{M \times U \times \mathop \smallint \nolimits_{0}^{t} I_{{\text{t}}} {\text{ d}}t}} } \\ \end{array} $$

3 $$ \begin{array}{*{20}c} {{\text{CE}} = \frac{{F \times \left( {C_{0} - C_{{\text{t}}} } \right) \times V_{{\text{s}}} }}{{M \times \mathop \smallint \nolimits_{0}^{t} I_{{\text{t}}} {\text{ d}}t}} \times 100\% } \\ \end{array} $$

4 $$ \begin{array}{*{20}c} {{\text{SRE}} = \frac{{C_{0} - C_{{\text{t}}} }}{{C_{0} }} \times 100\% } \\ \end{array} $$

5 $$ \begin{array}{*{20}c} {{\text{Productivity}} = \frac{{3600 \times V_{{\text{s}}} }}{A \times t}} \\ \end{array} $$

where *C*_0_ and *C*_t_ are the salt concentrations (g L^−1^) at initial and at time *t* (s), *V*_s_ is the volume of the saltwater (0.05 L), *M* is the molar mass of NaCl (58.69 g mol^−1^), A is the effective contact area between the flow electrode and the ion exchange membrane (16 cm^2^), *t* is the operation time (s), *I*_t_ is the transient current flowing through the FCDI system (*A*), *U* is the applied constant voltage (*V*) and *F* is the Faraday constant (96,485 C mol^−1^).

#### Computational Methods

The simulations based on finite element method (FEM) were conducted on COMSOL Multiphysics® Version 6.1 simulation software [34], which includes the computational fluid dynamics/particle tracking simulations and electrostatic field simulations. The detailed computational methods can be found in the Supplementary Note S3.2.

## Results and Discussion

### System Setup and Current Collector Design

**Fig. 1:**
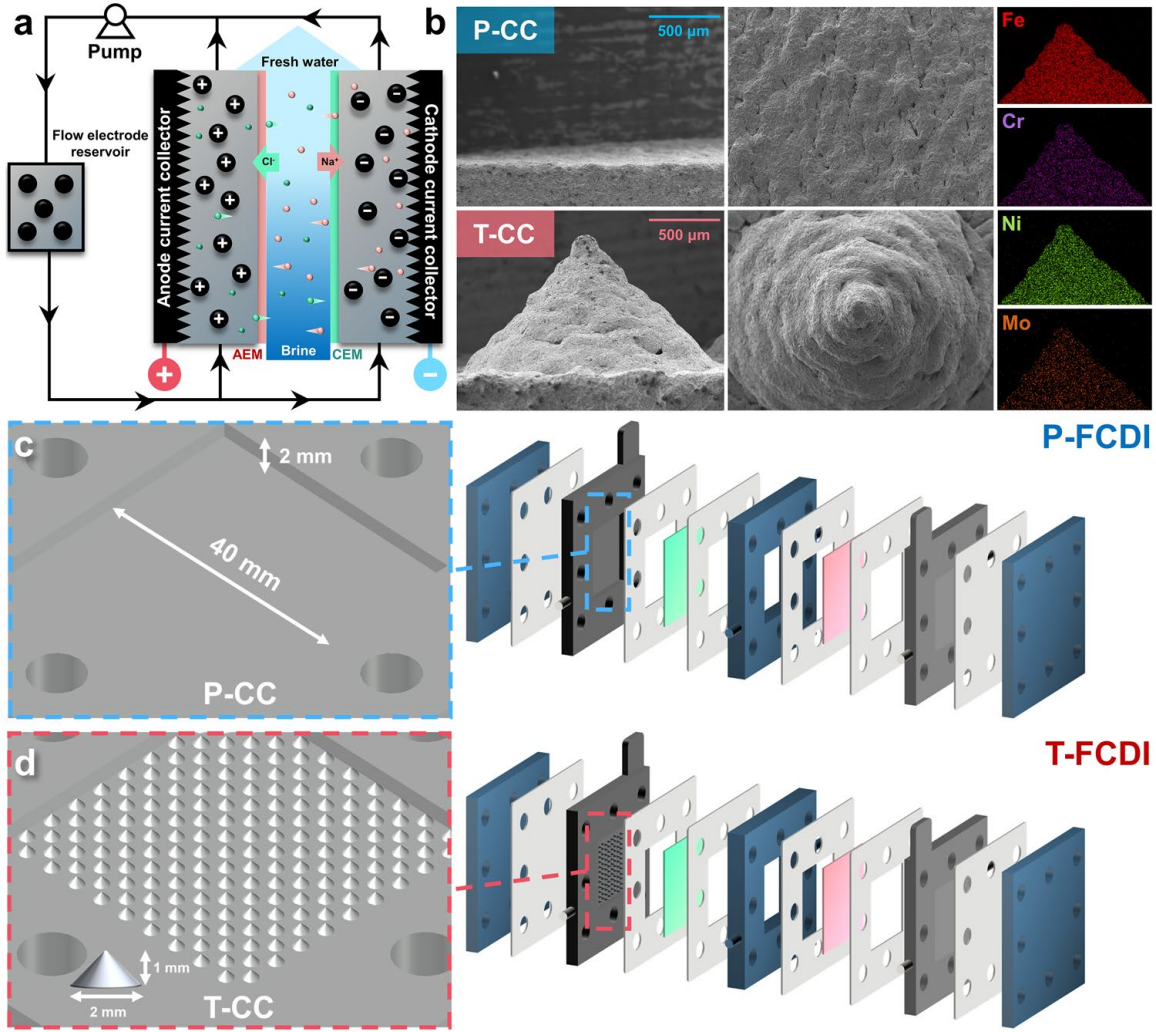
**a** System setup of the three-chamber FCDI system; **b** FE-SEM images and elemental mappings of the P-CC and T-CC; Schematics of the FCDI cells equipped with different current collectors: **c** P-FCDI, and **d** T-FCDI

The structure of our FCDI system is shown in Fig. 1a. When a constant voltage is applied to the FCDI system, anions and cations migrate through the anion exchange membrane (AEM) and cation exchange membrane (CEM), respectively, driven by the electric force. Subsequently, the ions are captured by the charged carbon particles, forming electrical double layer (EDL) on the carbon surface. The ion-containing carbon particles are then pumped and mixed in the external flow electrode reservoir to regenerate, where the charges are balanced, EDL is diminished, and the ions are released from the carbon particles into the solvent phase.

As the current collector plays a crucial role in FCDI systems, two types of current collectors made of 316 L stainless steel with good hydrophilicity (Fig. S4, detailed properties of the current collectors can be found in Table S4) were firstly fabricated using selective laser melting technique (XRD pattern shown in Fig. S5 confirms its austenite structure [35]), denoted as planar current collector (P-CC) and tip-array current collector (T-CC), respectively. Correspondingly, FCDI systems equipped with these current collectors are denoted as P-FCDI and T-FCDI. The micromorphology and the elemental distributions (indicating the presence of Fe, Cr, Ni, and Mo elements) of the current collectors are shown in Fig. 1b. The tips are cone-shaped with a bottom diameter of 2 mm and a height of 1 mm. It is worth noting that a smaller bottom diameter (thus a higher tip density) enables a higher performance according to our preliminary screening simulations but a 10 × 10 tip array structure (corresponding to a bottom diameter of 2 mm) is employed here due to the technical limit of metal 3D printing at this stage. The structures of the P-FCDI and T-FCDI are shown in Fig. 1c, d, where the current collector is the only difference between these two FCDI systems.

### Computational Simulations and Analysis

#### Computational Fluid Dynamics/Particle Tracking Simulations

**Fig. 2:**
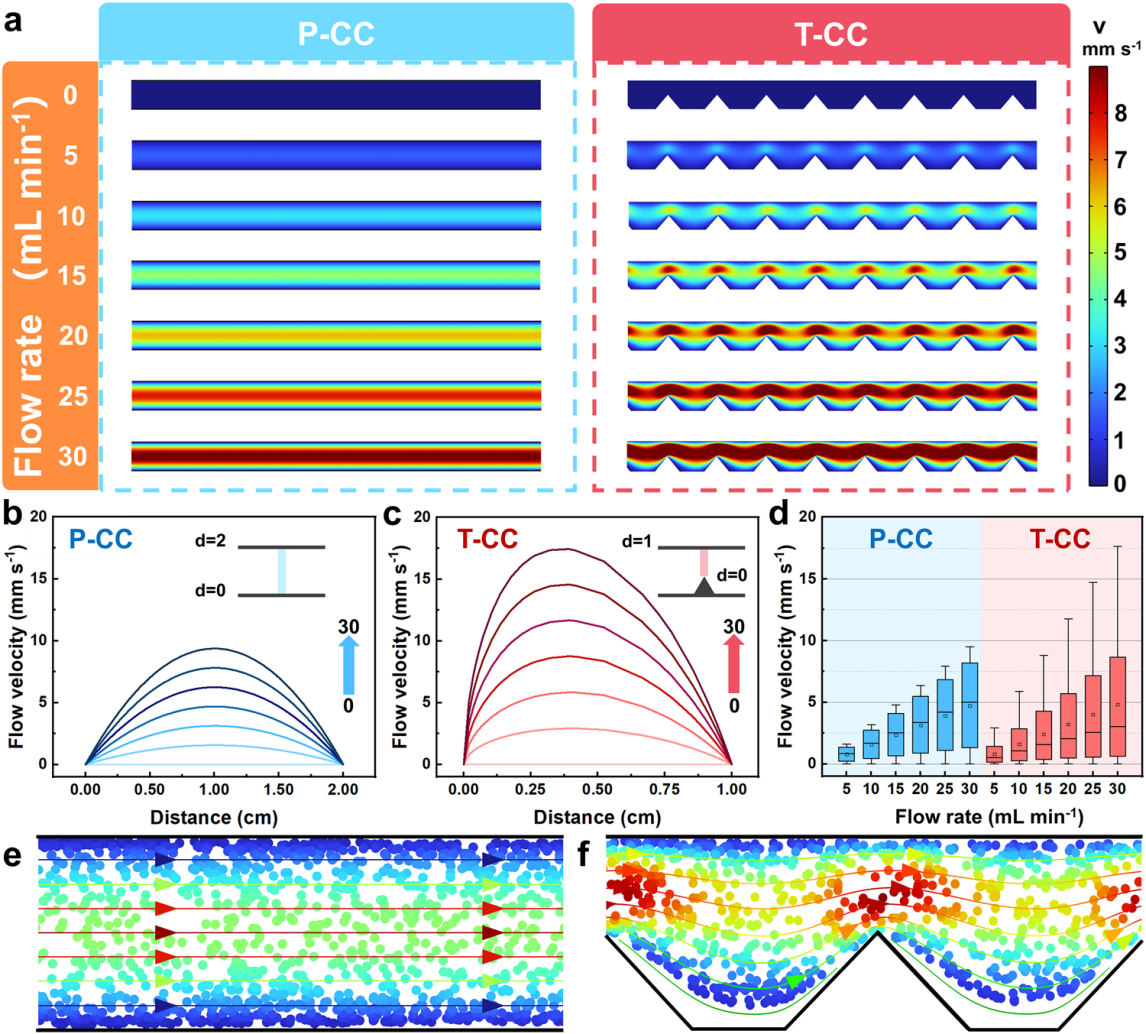
**a** Flow velocity distributions at different inlet flow rates in P-CC and T-CC; The velocity distributions in the cross-sectional direction for: **b** P-CC, and **c** T-CC; **d** Boxplots of the flow velocities for P-CC, and T-CC; Velocity distributions of the particles in: **e** P-CC, and **f** T-CC, in which the inlet flow rate was 15 mL min^−1^

The flow chamber geometry and the flow rate are two major factors influencing the flow patterns, thus exerting significant effects on the collision of particles and the formation of conductive network. To investigate the hydrodynamic characteristics of the flow electrode in different current collectors, we employed a computational fluid dynamics (CFD) model for the flow electrode with a solid content of 5 wt% [36], and simulated the velocity distributions under different inlet flow rates. The velocity distributions in the flow channels of the P-CC and T-CC at different flow rates are shown in Fig. 2a. Flow channels with both current collectors exhibit evident boundary layer effect, i.e.*,* the velocity adjacent to the wall is significantly lower than that in the middle of the flow channel, which is more clearly revealed in Fig. 2b, c. It is noted the possibility for particle sedimentation is higher and charge conduction is worse in the boundary layer [37]. In the P-CC, the flow electrode exhibits a regular laminar distribution without obvious turbulence. In contrast, the slurry velocity increases significantly in the T-CC when passing through the tips due to the narrowing of the channel. The boxplots of the flow velocity for both current collectors are shown in Fig. 2d, where the data were collected from all meshes in the FEM simulation results. In the boxplots, the dots are the average velocities, and the boxes contain the velocity data in the interquartile range (50% of the data set); the horizontal lines in the boxes are the middle values of the data set, the horizontal lines at the top and the bottom are the maximum and minimum values. It can be seen that T-CC can increase the average flow velocities at all flow rates. Interestingly, no significant changes were found in the interquartile range for both current collectors. However, the T-CC increases the flow velocity in the high-velocity range (velocity ranged at the top 25% of the data set), indicating the local flow field enhancement effect induced by T-CC (detailed data can be found in Table. S5). This was also confirmed by the in situ fluid transport and mixing experiments (results are shown in Fig. S6).

The velocities of the carbon particles were further tracked at an inlet flow rate of 15 mL min^−1^ to study the influence of current collector on the flow behavior (particle modeling was based on the super particle (SP) method, which can be found in the Supplementary Note 3.2 [38]). The velocity distributions of the carbon particles in the P-CC and T-CC are shown in Fig. 2e, f, and the video version can be found in the Online Resource (ESM_4-7.avi). For P-CC, particles in the boundary layer move relatively slow (the risk of sedimentation is thus relatively high), which flow much faster with sparser distribution in the middle of the flow channel. Therefore, particles in the P-CC only flow within a limited range of the flow layer, with weak particle–particle and particle-current collector interactions, which is unfavorable for the formation of the conductive network. In contrast, in the T-CC, particles move in a wave-like manner (Fig. 2f). For the particles in the boundary layer, after colliding with the current collector and acquiring charges, they immediately move away from the wall under the influence of the reverse momentum and fluid drag, resulting in more frequent particle collisions in the direction perpendicular to the flow. Thus, the tip structures effectively transfer the charged particles in the boundary layer and enhance the interactions of the particles perpendicular to the flow direction. On the one hand, this alleviates the excessive accumulation of the carbon particles in the boundary layer, thus promoting charge permeation. On the other hand, it fully utilizes the carbon particles in the electrode chamber (i.e., increasing the equivalent flow electrode solid content), which enables higher desalination performances.

#### Electric Field Simulation

**Fig. 3:**
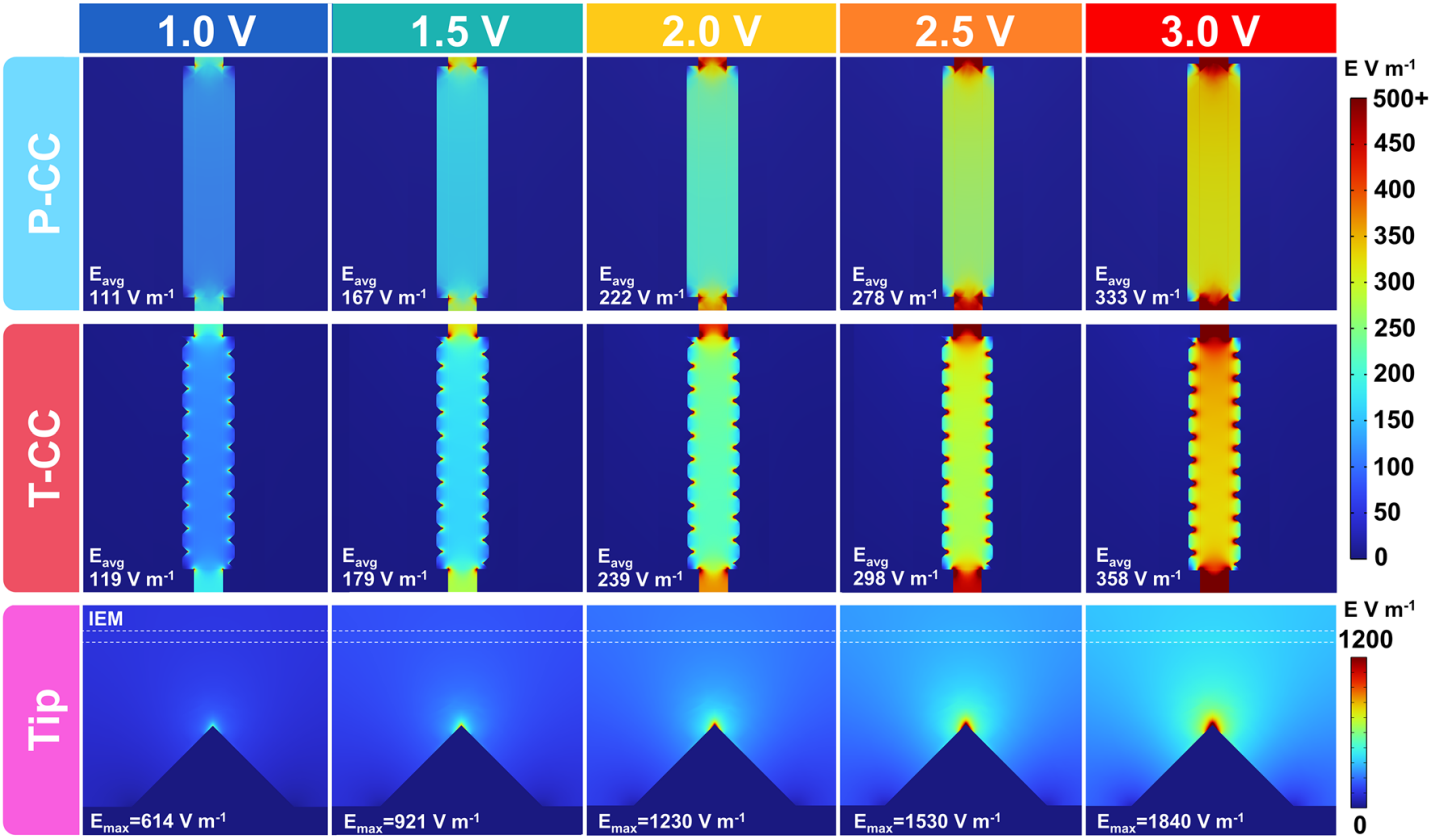
Simulation results of the electric field distribution in P-FCDI, T-FCDI, and the tip of the T-CC

By using the electrostatic field simulation model [39] (detailed computational methods are described in the Supplementary Note S3.2), we simulated the electric field distributions of the FCDI cells equipped with P-CCs and T-CCs at different voltages (Fig. 3). The simulation results indicate that the electric field intensity increases with the voltage for both types of FCDI cells. At all voltages investigated, the electric field distribution in P-FCDI exhibits a uniform and parallel distribution. With the voltage increased from 1.0 to 3.0 V, the average electric field intensity within the P-FCDI increases from 113 to 333 V m^−1^. In comparison, the T-FCDI exhibits significant electric field enhancements near the tips. With the increase in voltage from 1.0 to 3.0 V, the average electric field intensity within the T-FCDI increases from 119 V m^−1^ at 1.0 V to 358 V m^−1^ at 3.0 V. Most strikingly, the maximum electric field intensity at the tip reaches up to 614 V m^−1^–1.0 V, approximately 5.2 times higher than the electric field intensity in the plane region. At 3.0 V, the electric field intensity at the tip reaches 1840 V m^−1^, and the enhanced electrical field extends to the surface of the ion exchange membrane, thus providing higher driving forces for ion migration.

**Fig. 4:**
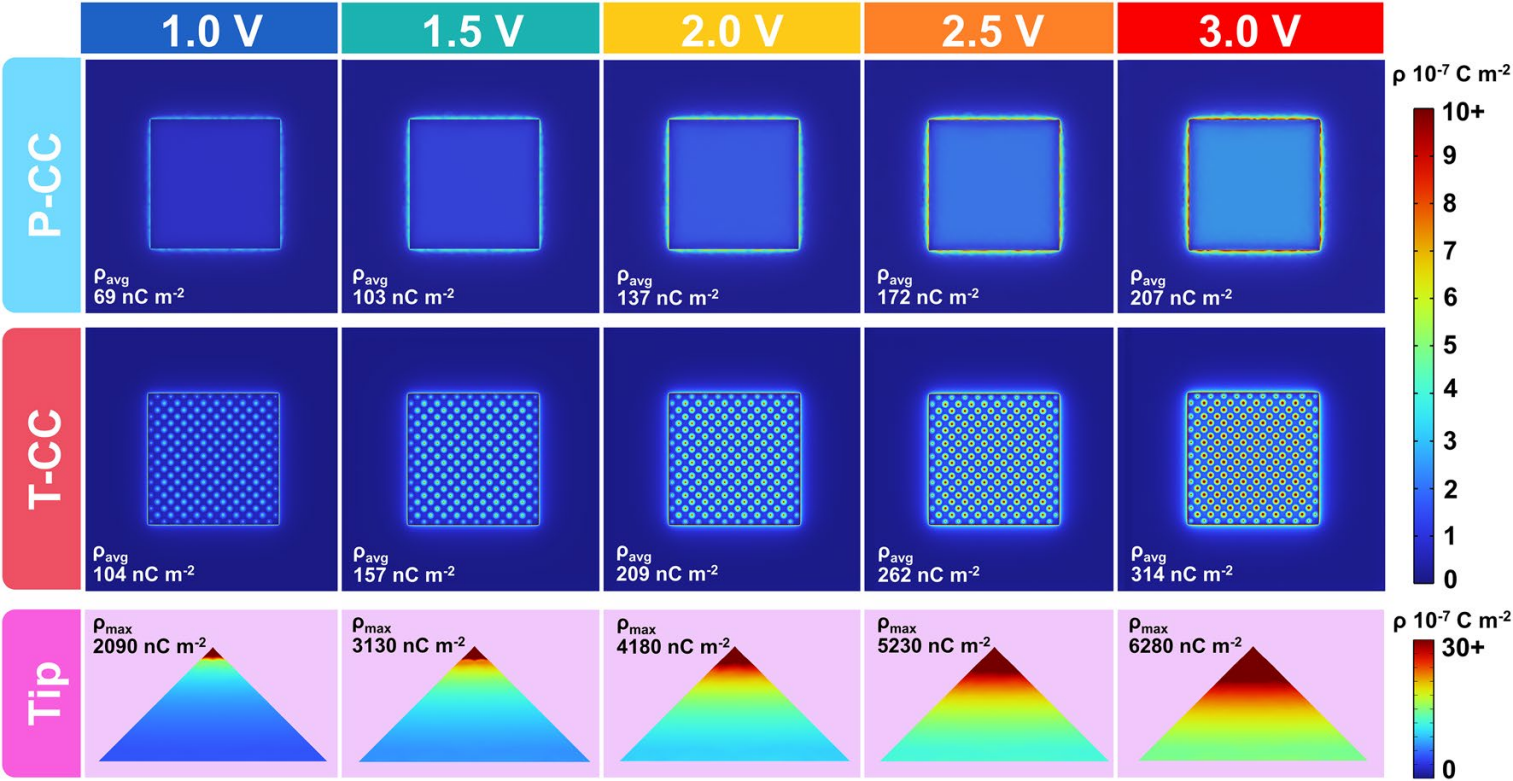
Simulation results of the surface charge density distribution in P-FCDI, T-FCDI, and the tip of the T-CC

Additionally, the simulation results suggest that the surface charge density distribution is uniform in P-CC. The charge density gradually increases with the increase in voltage, but remains uniformly distributed (Fig. 4). With the voltage increased from 1.0 to 3.0 V, the average surface charge density of the P-FCDI increases from 69 to 207 nC m^−2^. Again, the T-FCDI exhibits a noticeable surface charge density enhancement near the tip. The average electric field intensity within T-FCDI from 104 nC m^−2^ at 1.0 V to 314 nC m^−2^ at 3.0 V. On the surface of the T-CC, the charge density distribution exhibits a volcano shape (the charge density is higher at the tip and decreases as the curvature decreases). This suggests that the localized electric field enhancement effect is due to the enrichment of charges at the tip where the curvature of the conductor surface is maximized. The tip effect provides a greater electric field driving force for desalination at lower operating voltages, thereby accelerating ion migration and charge transfer kinetics of the FCDI system.

### Electrochemical Impedance Spectroscopy with Distribution of Relaxation Times Analysis

To investigate the electrochemical characteristics of the FCDI systems equipped with different collectors, electrochemical impedance spectra were acquired and distribution of relaxation times (DRT) analysis was employed to decouple impedance data (dots shown in Fig. S7a, b). Differential of the spectra into a series of parallel resistor-capacitance (RC) circuits gave rise to the DRT plot (as shown in Fig. S7c, in which the inset magnifies the high relaxation time region). It is worth noting that all impedance data were obtained under semi-equilibrium conditions and were validated through Kramers–Kronig validity tests (Fig. S7d) [32].

**Fig. 5:**
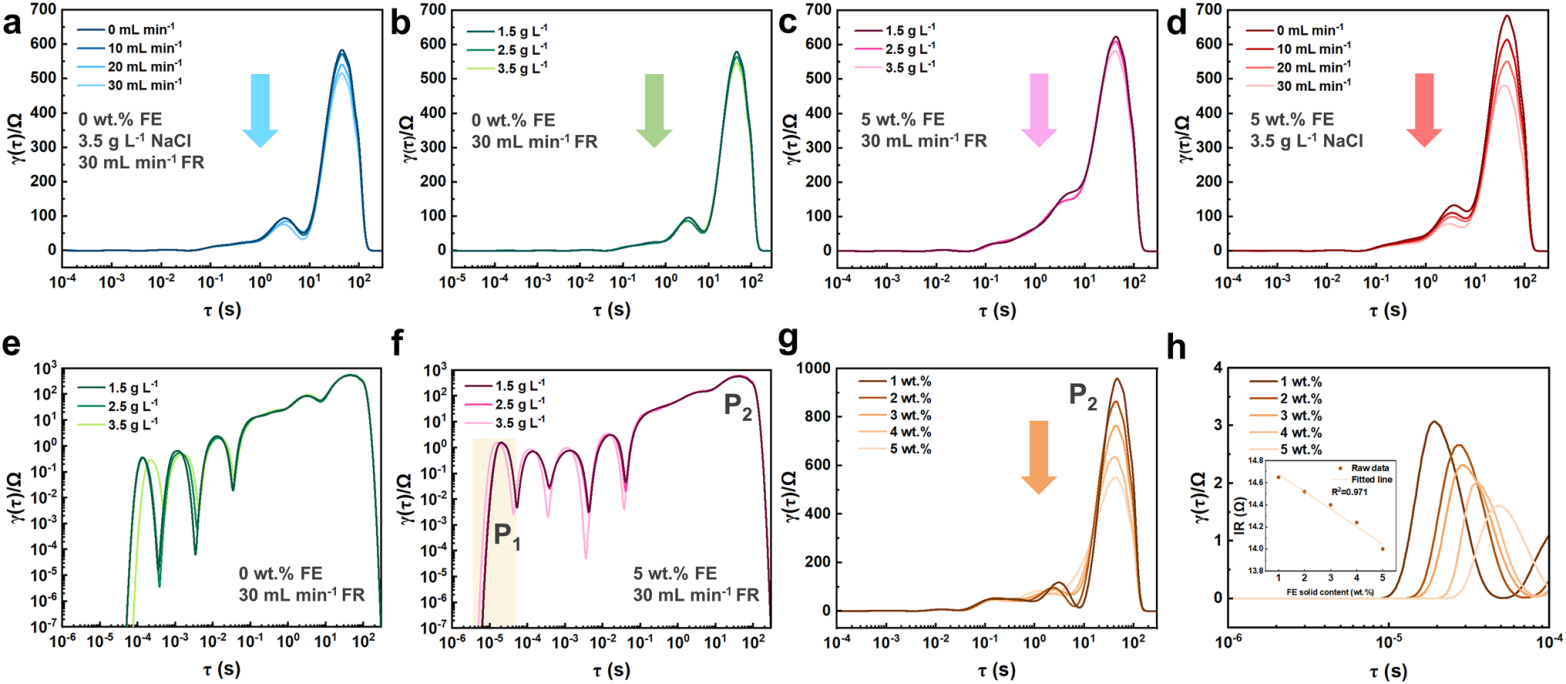
DRT plots obtained at various conditions: **a** effect of flow rate of saltwater, **b** effect of salt concentration (the solid content in FE was 0%), **c** effect of salt concentration (the solid content in FE was 5%), and **d** effect of flow rate of flow electrode; Logarithmic transformed DRT plots for: **e** ED system, and **f** FCDI system with different salt concentrations; DRT plots magnified at different time regions for: **g** high relaxation time region, and **h** low relaxation time region in which the inset is the dependence of internal resistance on the solid content

Before investigating the kinetic differences between different current collectors via electrochemical impedance spectroscopy with distribution of relaxation times analysis (EIS-DRT), it is necessary to identify different peaks appeared in the DRT plots. Firstly, we obtained impedance spectra from an FCDI system (equipped with two T-CCs in this section) containing 0 wt% of solid in flow electrode (i.e., an ED system). The salinity of the saltwater was fixed at 3.5 g L^−1^ with its flow rate varied from 0 to 30 mL min^−1^, whereas the flow rate of the flow electrode was fixed at 30 mL min^−1^. The obtained DRT plots are shown in Fig. 5a. The intensity of the peak with a relaxation time of c.a. 40 s, which has the longest relaxation time and the highest intensity compared to others, decreases with the flow rate of the saltwater. Therefore, this peak can be attributed to the ion transport process [40]. The thickness of the diffusion layer near the ion exchange membranes (IEMs) decreases with the increase in flow rate, thus alleviating the concentration polarization at the IEMs surface [41, 42]. DRT plots for the ED system and the FCDI system (solid content in the flow electrode was 5 wt%) were then obtained with different salinity, as shown in Fig. 5b, c. The DRT plots at the high relaxation time region show the same trend as Fig. 5a, indicating that a higher salt concentration leads to a lower ion transport barrier. Subsequently, the DRT plots of the FCDI system with different flow rates of the flow electrode were obtained and shown in Fig. 5d, in which the ion transport barrier decreases as the flow rate increases. Compared to Fig. 5a, the flow rate of the flow electrode has a greater impact on the ion transfer barrier than that of the saltwater. Therefore, it can be concluded that the ion transfer process can be enhanced with the addition of the carbon particles.

Moreover, the system’s internal resistance (IR) was evaluated from the EIS data at high-frequency region [40]. As shown in Fig. S8a, b, for the ED system, the internal resistance decreases with the increase of flow rate or salt concentration. While for the FCDI system, a higher salt concentration also leads to a lower internal resistance (Fig. S8c). Moreover, the decreasing rates of the internal resistance as a function of concentration for the two systems are similar, i.e., − 3.485 Ω L g^−1^ for ED system and − 3.715 Ω L g^−1^ for FCDI system. However, an opposite trend is induced by the flow electrode, i.e.*,* the internal resistance increases with the flow rate of the flow electrode (Fig. S8d). This agrees well with the CFD simulation results that an increasing flow electrode flow rate leads to a more significant boundary layer effect, which makes the charge transfer process more difficult and resulting in a higher internal resistance.

Logarithmic transformation of the DRT data reveals a whole picture of the electrochemical processes involved in the electrochemical system. As shown in Fig. 5e, for an ED system, three small peaks with relaxation time of c.a. 10^–4^, 10^–3^, and 10^–2^ s are attributed to the charge transfer process at the current collectors/electrolyte interface, the corrosion reaction of the current collectors and the gas evolution reactions (*i.e*., water splitting and chlorine evolution reaction) [43–45], respectively. The overlapped peaks located in the range of 10^–2^–10^1^ s are related to the EDL formation processes. Importantly, a new peak appears in the short relaxation time region of the DRT plots obtained from the FCDI system (Fig. 5f). It is reasonable to attribute this peak to the charge transfer process of the flow electrode as this peak is only observed in the FCDI system (whereas it is absent in the ED system). We further validated this hypothesis by conducting EIS-DRT analysis to FCDI systems with different solid contents in the flow electrode. As shown in Fig. 5g, a higher flow electrode solid content leads to a lower ion transport barrier. Meanwhile, the charge transfer barrier shown in the low relaxation time region of the DRT plots (Fig. 5h) and the system internal resistance (inset in Fig. 5h) continuously decreases with the increase of solid content in flow electrode. This can be understood that a higher solid content can form a conductive network with improved connection and integrity.

**Fig. 6:**
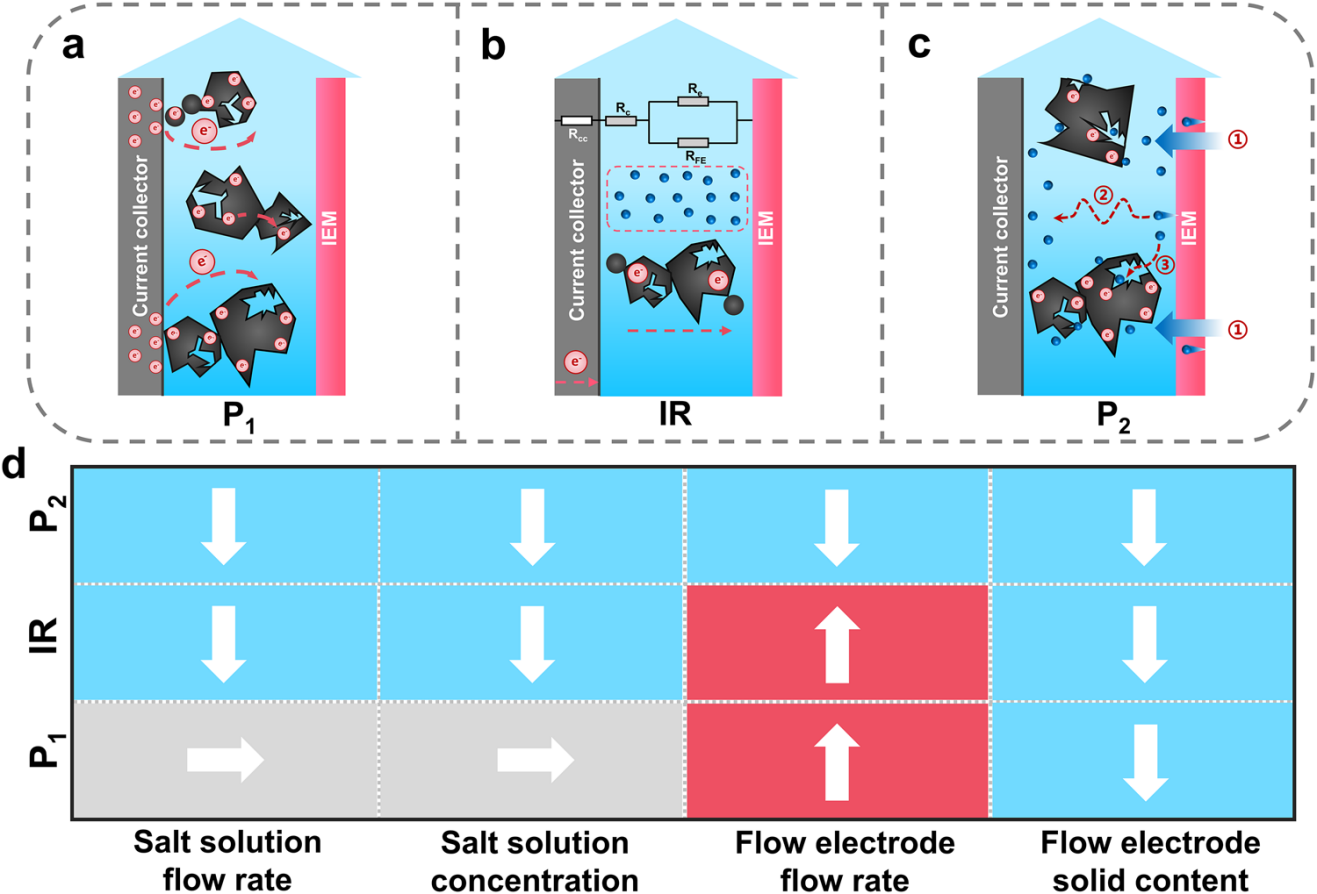
Schematics of: **a** charge transfer process of the flow electrode, **b** system internal resistance, and **c** ion transport process; **d** Dependence of the intensity of P_1_ (P_2_) and the value of IR on the operation condition. The up arrow and the down arrow refer to the positive correlation and negative correlation, and the horizontal arrow indicates that there is no obvious dependence between two variables

It is thus unveiled that the peak with the lowest relaxation time (P_1_) is attributed to the charge-transport process of the flow electrode (Fig. 6a). The system’s internal resistance is considered as the total equivalent resistance of the system (Fig. 6b). The equivalent circuit model (inset in Fig. 6b) consists of the current collector resistance (*R*_cc_), contact resistance between the flow electrode and the current collector (*R*_c_), background electrolyte resistance (*R*_e_), and the flow electrode resistance (*R*_FE_). The peak with the longest relaxation time (*P*_2_) is attributed to the ion transport process (Fig. 6c), which can be divided into three processes: (1) transmembrane ion transport, (2) ion transport in the electrolyte, and (3) ion transport in the carbon pores. The variation tendencies for the intensity of *P*_1_ and *P*_2_, and the value of internal resistance (IR) when the corresponding operational condition increases are summarized in Fig. 6d. It is expected that a lower peak intensity of *P*_1_ and *P*_2_, and a lower value of internal resistance could lead to a higher desalination performance.

**Fig. 7:**
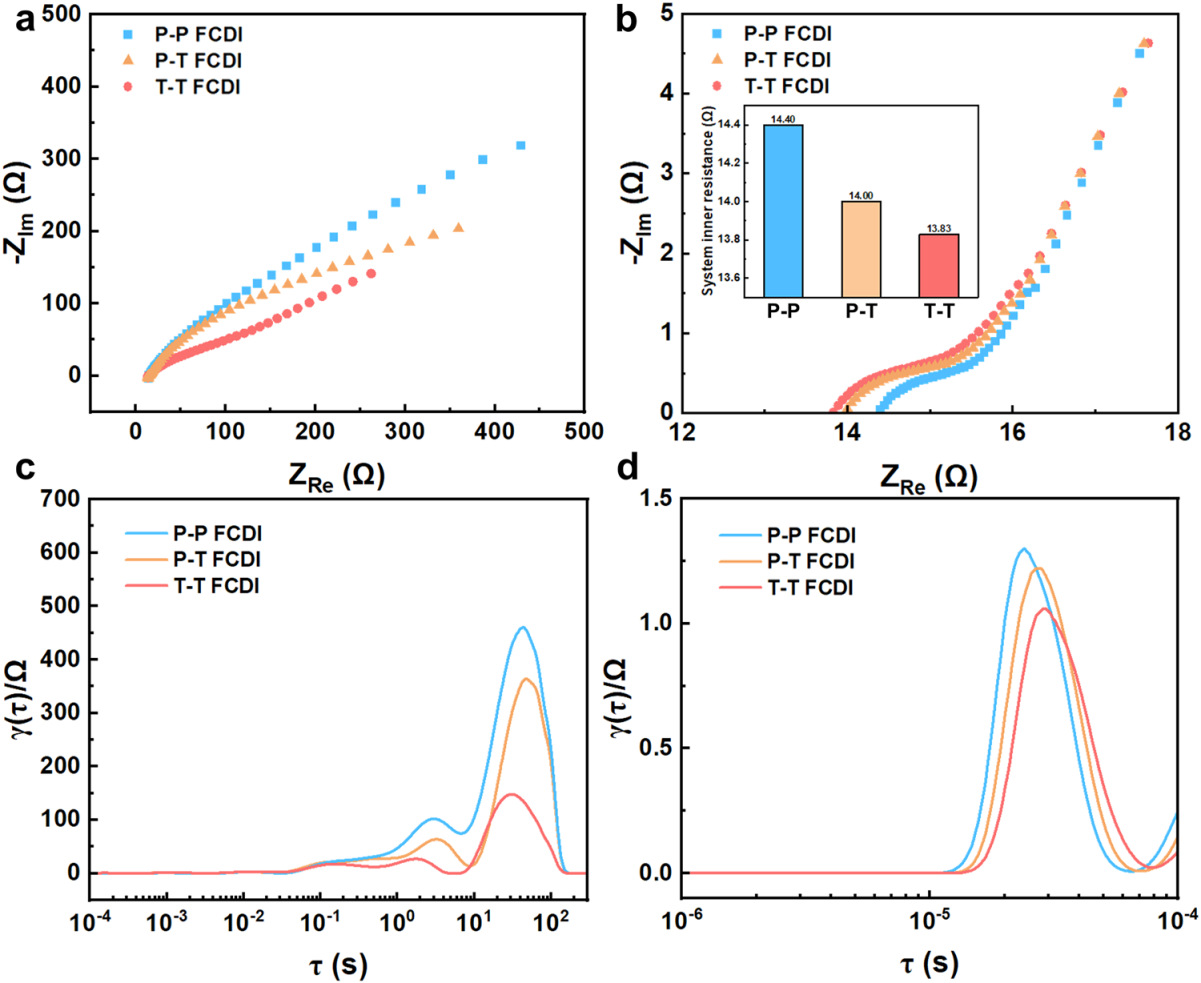
**a** Full frequency, and **b** high-frequency region of the Nyquist plots; DRT plots in: **c** high relaxation time region, and **d** short relaxation time region obtained from the P-P, P–T, and T-T combinations

To investigate the promotion effect of the T-CC, different combinations of current collectors for the two-electrode FCDI systems were constructed, i.e.*,* two P-CCs for both negative and positive electrodes (denoted as P-P FCDI), one P-CC and one T-CC (denoted as P–T FCDI), and two T-CCs (denoted as T-T FCDI). Nyquist plots for these systems are shown in Fig. 7a. It is obvious that the internal resistance of T-T FCDI (13.83 Ω) is lower than P–T FCDI (14.00 Ω) and P-P FCDI (14.40 Ω), as can be seen from the high-frequency region in the Nyquist plots (Fig. 7b). The DRT plots obtained from these systems are displayed in Fig. 7c, d. It can be seen the T-T combination shows the lowest ion transport barrier and the flow electrode charge transfer barrier, which confirms the optimal flow field and electric field endowed by the T-CCs.

### Desalination Performance

**Fig. 8:**
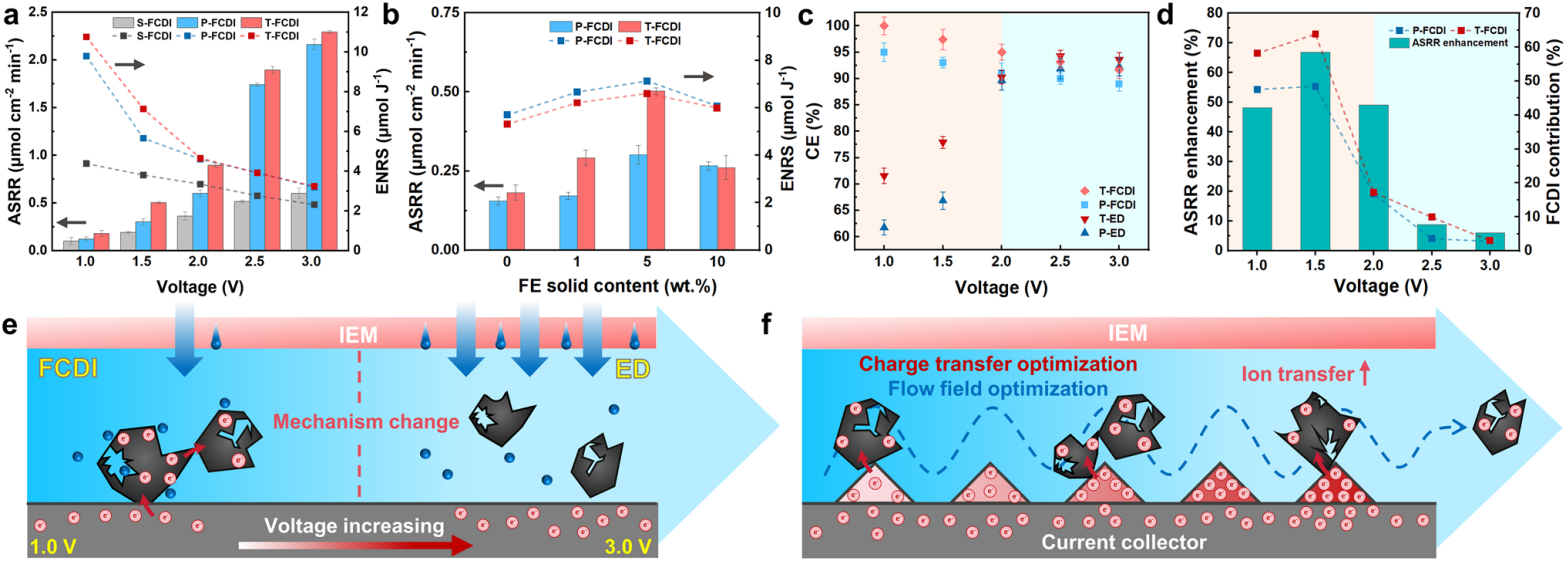
**a** ASRR, ENRS for S-FCDI, P-FCDI and T-FCDI; **b** ASRR and ENRS for T-FCDI with different solid contents in the flow electrode (FE); **c** CE comparison plots of the ED and FCDI systems equipped with different current collectors; **d** ASRR enhancement of T-FCDI and FCDI contribution of P-FCDI and T-FCDI; **e** Schematic of the voltage-dependent ion transport mechanism in FCDI; **f** Schematic of the T-FCDI enhancement mechanism

The desalination performance of P-FCDI, T-FCDI and S-FCDI (*i.e.*, an FCDI cell equipped with conventional current collectors and serpentine flow channels) was first evaluated with a solid content of 5 wt% and a saltwater salinity of 3.5 g L^−1^. Unsurprisingly, T-FCDI shows the highest average salt removal rate (ASRR) and energy normalized removed salt (ENRS) at all voltages (Fig. 8a). Specifically, at 1.5 V, the ASRR of the T-FCDI is 0.50 μmol cm^−2^ min^−1^, which is 1.67 folds and 2.65 folds higher than that of P-FCDI (0.30 μmol cm^−2^ min^−1^) and S-FCDI (0.19 μmol cm^−2^ min^−1^). The ENRS of the T-FCDI operated at 1.5 V is 7.12 μmol J^−1^, which is 1.26 folds and 1.87 folds higher than that of P-FCDI (5.65 μmol J^−1^) and S-FCDI (3.80 μmol J^−1^).

The charge efficiency (CE) refers to the proportion of the charge involved in ion removal to the total charge, which can be expressed as:

6 $$ \begin{array}{*{20}c} {{\text{CE}} = \frac{{Q_{{\text{I}}} }}{{Q_{{\text{I}}} + Q_{{\text{F}}} + Q_{{\text{D}}} }} \times 100\% } \\ \end{array}$$

where the total charge consists of three components: ion transport charge (*Q*_I_), faradaic reaction charge (*Q*_F_) and the dissipation charge (*Q*_D_). Herein, *Q*_I_ refers to the transmembrane ion flux from the saltwater chamber to the flow electrode chamber. This proportion can be described by the Nernst–Planck (NP) equation [46]. *Q*_F_ is attributed to all faradic reactions that occurred in the FCDI system, which can be expressed by the generalized Frumkin–Butler–Volmer (gFBV) equation [47]. *Q*_D_ is the dissipated charge due to neutralization of negative and positive electrodes. Charge dissipation mainly occurs in the following ways: carbon particles obtained electrons from the current collectors or the surrounding carbon particles directly flow into the external flow electrode reservoir and neutralize their charges without participating in capacitive adsorption (see Supplementary note 3.3 for detailed explanations).

As shown in Fig. S9a, for the FCDI system, the CE decreases with the increase of voltage. This can be understood that the ion transport charge (*Q*_I_, based on NP equation) and the charge contributed by the Faradaic reactions (*Q*_F_, based on gFBV equation) both increase with the increase in voltage (evidenced by LSV results and XPS data, see Figs. S10 and S11). However, it is reasonable to assume that for the FCDI system, the increase of *Q*_I_ is less than that of *Q*_F_, which indicates that FCDI is more suitable for operation at low voltages. The contradiction is that in order to ensure the desalination rate, it is necessary to appropriately increase the voltage and sacrifice parts of the *Q*_F_ to achieve more *Q*_I_. Among all FCDI systems, T-FCDI demonstrates the highest CE, which can be explained by the following three reasons: First, T-CC can enhance the local electric field intensity. According to NP equation, this can increase the driving force of ion migration, thereby enhancing ion transport (*i.e.,* increasing *Q*_I_); Second, T-CC optimizes the electric field and flow field, promoting charge transfer between the current collector and the carbon particles, thereby avoiding electron accumulation on the surface of the current collector and depressing the faradic reactions (*i.e*., decreasing *Q*_F_); Third, electrons can be evenly distributed on the surface of the carbon particles, increasing the local electric field intensity on the surface of the carbon particles. According to the modified Donnan (mD) model [48], the increase in the electrostatic potential on the surface of the carbon particles can increase the ion storage capacity per particle, thereby improving the ion transport kinetics (*i.e.,* increasing *Q*_I_).

The effect of the flow electrode solid content on the performance was further investigated (Figs. 8b and S9b). The ASRR, ENRS and CE all increase with the flow electrode solid content increase and reach their maximum as the flow electrode solid content is 5 wt%, beyond which these metrics all decrease. At a low flow electrode solid content, the 3D conductive network is insufficient, which leads to poor charge transfer and ion transport kinetics. When the flow electrode solid content increases, the conductive network is improved, which is responsible for enhancing the desalination performance. However, flow electrode sedimentation and congestion occur when the flow electrode solid content is higher than the optimal value (Fig. S12). As such, the charges carried in the carbon particles may not be balanced by the counterions and participate in ion transport (*i.e.,* contribute to *Q*_I_). Instead, carbon particles directly flow into the external flow electrode reservoir and balance their own charges. This could lead to an increase in dissipation charge (*Q*_D_), resulting in a lower CE.

To further understand the enhancement mechanism of the T-FCDI, the ASRR (Fig. S9c) and ENRS (Fig. S9d) of four systems were evaluated: P-ED (*i.e.,* an ED system equipped with P-CCs), T-ED (*i.e.,* an ED system equipped with T-CCs), P-FCDI and T-FCDI. Under all conditions, the FCDI systems show higher ASRR and ENRS than ED systems. Additionally, the systems equipped with T-CCs show higher ASRR and ENRS than those equipped with P-CCs. This is due to the enhanced flow field and electric field caused by T-CCs. As shown in Fig. 8c, there are significant differences for the voltage dependence of CE for ED and FCDI systems. As the voltage increases, the CE for both FCDI systems decreases, whereas for ED systems, the CE gradually increases to *c.a.* 90% and then drops slightly when the voltage is over 2.5 V. The reason for this phenomenon (similar results was reported in Refs. [49] and [50]) is that the increasing magnitude of *Q*_I_ and *Q*_F_ with voltage are different. At low voltages (e.g., below 2.5 V), increasing the voltage results in a greater increase of *Q*_I_ than *Q*_F_, thus exerts a positive effect on CE. The relative change of *Q*_I_ and *Q*_F_ at higher voltages (e.g., above 2.5 V) is responsible for the reversed trend shown in Fig. 8c.

The ASRR enhancement (calculated as: ASRR_T-FCDI_-ASRR_P-FCDI_)/ASRR_P-FCDI_) and the FCDI contribution (calculated as: ASRR_FCDI_-ASRR_ED_)/ASRR_FCDI_) at different voltages are shown in Fig. 8d. The results indicate that as the voltage increases, the ASRR enhancement first increases and then declines, reaching the maximum at 1.5 V (66.9%), and becoming negligible at 2.5 V (8.8%) and 3 V (6.0%). The variation trend of the FCDI contribution displays a similar behavior to that of the ASRR enhancement, reaching the maximum at 1.5 V (63.9% for T-FCDI and 48.5% for P-FCDI). It is reasonable to conclude that: (i) the ion transport mechanism of the FCDI system is voltage-dependent (Fig. 8e). At low voltages, the potential gradient for ion migration is relatively low, and the electrodialysis contribution is insignificant and capacitive adsorption process is dominating (thus showing an FCDI type of ion-capturing). At higher voltages, the FCDI system can be considered as the same as the ED system (thus showing an ED mechanism), where slight enhancements can be found due to the improved conductivity caused by the flow electrode; (ii) The enhancement effect of the T-CCs is valid mainly on the FCDI mode (rather than on ED mode). The role of T-CCs in optimizing the flow field and charge transfer of the flow electrode outweighs their enhancement of ion transport due to the electric field enhancement effect (Fig. 8f).

**Fig. 9:**
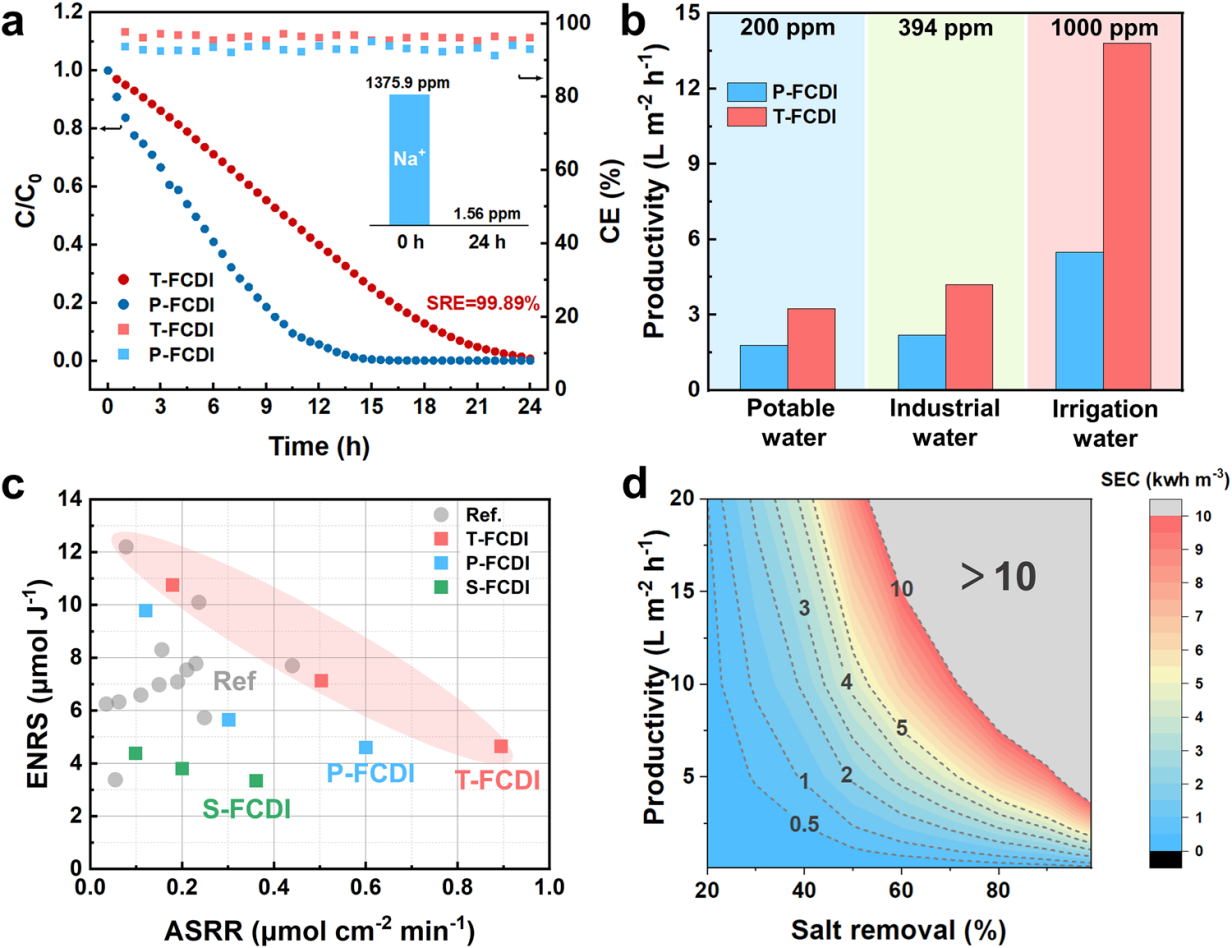
**a** Concentration variation profiles of the saltwater and CE profiles of the P-FCDI and T-FCDI. The inset is the Na^+^ concentration of the saltwater before and after 24 h of desalination test; **b** Productivity of the FCDI systems for different applications; **c** Performance comparison plots of the S-FCDI, P-FCDI and T-FCDI with literature data; **d** Specific energy consumption (SEC) for T-FCDI as a function of salt removal efficiency and productivity

Furthermore, to evaluate the long-term stability of the T-FCDI system, 24 h of continuous desalination tests were conducted. As shown in Fig. 9a, as the desalination process proceeded, the concentration of the saltwater continuously decreased. The salt concentration decreased from 3.5 g L^−1^ (Na^+^ concentration of 1375.9 ppm) to 3.85 mg L^−1^ (Na^+^ concentration of 1.56 ppm), corresponding to a salt removal efficiency (SRE) of 99.89% (Fig. 9a). The CE of the T-FCDI system remains above 95% during the whole test. Besides, we conducted 7 days of continuous operation and an ASRR retention of 90% was achieved (Fig. S13), which confirms its superior long-term stability. Furthermore, the productivity of the P-FCDI and T-FCDI under different standards are shown in Fig. 9b. Herein, the potable water complies with the WHO standard (*i.e.,* Na^+^ concentration below 200 ppm) [51], the industrial water (below 394 ppm) and irrigation water (below 1000 ppm) standards comply with the national standard of China (GB 5084-2021 and GB/T 19923-2005). The productivities of the T-FCDI system for potable water, industrial water and irrigation water are 3.25, 4.19, and 13.82 L m^−2^ h^−1^, respectively, which are 1.82, 1.91, and 2.51 times higher than that of P-FCDI. These results demonstrate the great potential of T-FCDI for desalination of brackish water.

The performance of the T-FCDI developed in this work is comparable or even superior to the leading results of the FCDI systems for NaCl desalination reported so far (Fig. 9c, details can be found in Table S6). As the productivity or the salt removal efficiency increases, the specific energy consumption (SEC) of the FCDI system increases, as shown in Fig. 9d. It can be seen that the T-FCDI is particularly favorable for desalinating brackish water for irrigation water or saline‐alkali land amendment (i.e., high productivity but low salt removal efficiency). Besides, due to the low cost, small size and easy management features of the T-FCDI system [52], T-FCDI is also a promising candidate for personal or household water treatment equipment or portable water treatment device (i.e.*,* high salt removal efficiency but low productivity). By using membrane stack configuration, it is possible to achieve even higher energy efficiency and productivity [53, 54].

## Conclusions

In this study, a stainless-steel current collector with tip arrays structure for FCDI system was 3D-printed by the selective laser melting technology. Computational fluid dynamics and particle tracking simulations showed that the T-CC could effectively alleviate boundary effects and enhance local flow velocity. Meanwhile, electric field simulations suggested that the tip structure could increase the local electric field intensity and local surface charge density. Electrochemical impedance spectroscopy with distribution of relaxation times analysis revealed that T-FCDI owned the lowest ion transport barrier, flow electrode charge transfer barrier and system internal resistance. Desalination tests results showed that at a condition of 5 wt% flow electrode solid content, 1.5 V operating voltage, and an influent of 3.5 g L^−1^ NaCl solution, the ASRR and ENRS of T-FCDI were 0.50 μmol cm^−2^ min^−1^ and 7.12 μmol J^−1^, which were 2.65 folds and 1.87 folds higher than that of the traditional FCDI system equipped with serpentine current collectors. Furthermore, we found that the ion transport mechanism of FCDI is voltage-dependent, primarily involving capacitance adsorption at low voltages and ion migration at high voltages. The tip-enhanced effect acts on the former, where the locally enhanced flow field and electric field promote both charge transfer and ion transport, thereby enhancing the desalination performance. Finally, 24 h of continuous desalination tests were performed. A salt removal efficiency of 99.89% was achieved, and the charge efficiency remained above 95% during the whole test for T-FCDI, confirming its superior long-term stability. The tip-enhanced FCDI system effectively promotes the desalination performance, which could be a promising candidate for personal/household water treatment equipment or portable water treatment devices in the future.

## Supplementary Information

Below is the link to the electronic supplementary material.

Supplementary file1 (DOCX 6452 KB)

Supplementary file2 (AVI 34 KB)

Supplementary file3 (AVI 438 KB)

Supplementary file4 (AVI 64 KB)

Supplementary file5 (AVI 327 KB)
