# Supplementary material for: Locally Enhanced Flow and Electric Fields Through a Tip Effect for Efficient Flow-Electrode Capacitive Deionization
Source: Nanomicro Lett. 2024 Sep 27;17:26. doi: 10.1007/s40820-024-01531-0 (PMC11436671; doi:10.1007/s40820-024-01531-0)
Supplement: Supplementary file 1 — Supplementary file1 (DOCX 6452 KB) [file 40820_2024_1531_MOESM1_ESM.docx]

Supporting Information for

**Locally Enhanced Flow and Electric Fields Through a Tip Effect for Efficient Flow-Electrode Capacitive Deionization**

Ziquan Wang^1,#^, Xiangfeng Chen^1,#^, Yuan Zhang^1^, Jie Ma^2^, Zhiqun Lin^3^, Amor Abdelkader^4,5^, Maria-Magdalena Titirici^6^, Libo Deng^1,^*

^1^College of Chemistry and Environmental Engineering, Shenzhen University, Shenzhen 518060, P. R. China

^2^Research Center for Environmental Functional Materials, College of Environmental Science and Engineering, Tongji University, 1239 Siping Road, 200092 Shanghai, P. R. China

^3^Department of Chemical and Biomolecular Engineering, National University of Singapore, Singapore 117585, Singapore

^4^Department of Engineering, Faculty of Science and Technology, Bournemouth University, Talbot Campus, Fern Barrow, Poole, England, BH12 5BB, UK

^5^Institut de Chimie de Nice, Université Côte d’Azur, UMR CNRS 7272, 28 Av. Valrose, 06108 Nice, France

^6^Department of Chemical Engineering, Imperial College London, South Kensington Campus, Exhibition Rd, London, SW7 2AZ, UK

# Ziquan Wang and Xiangfeng Chen contributed equally to this work.

*Corresponding author. E-mail: [Denglb@szu.edu.cn](mailto:Denglb@szu.edu.cn) (Libo Deng)

**S1 Supplementary Figures**


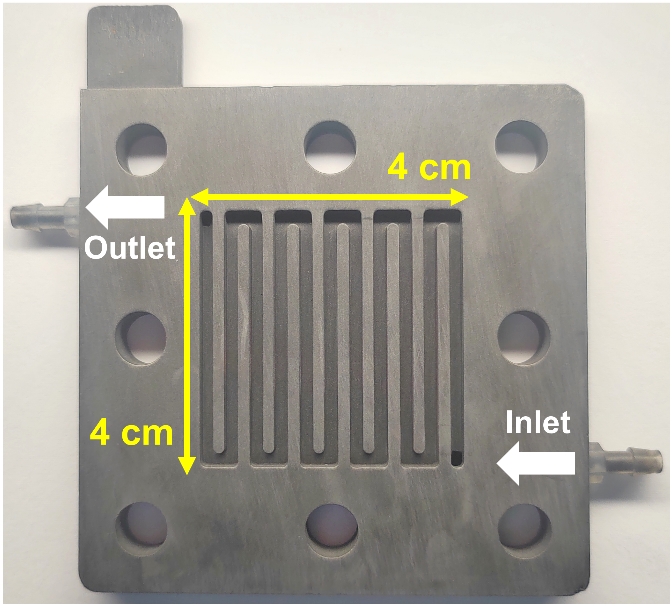


**Fig. S1** Digital image of the S-CC (traditional current collector)


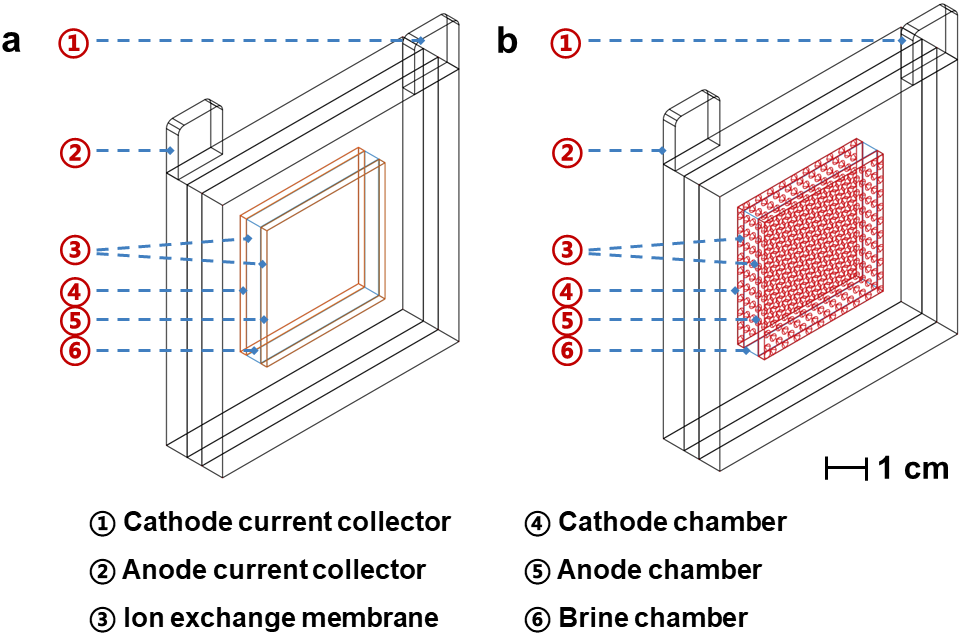


**Fig. S2** Structure diagram of: **(a)** P-FCDI and **(b)** T-FCDI


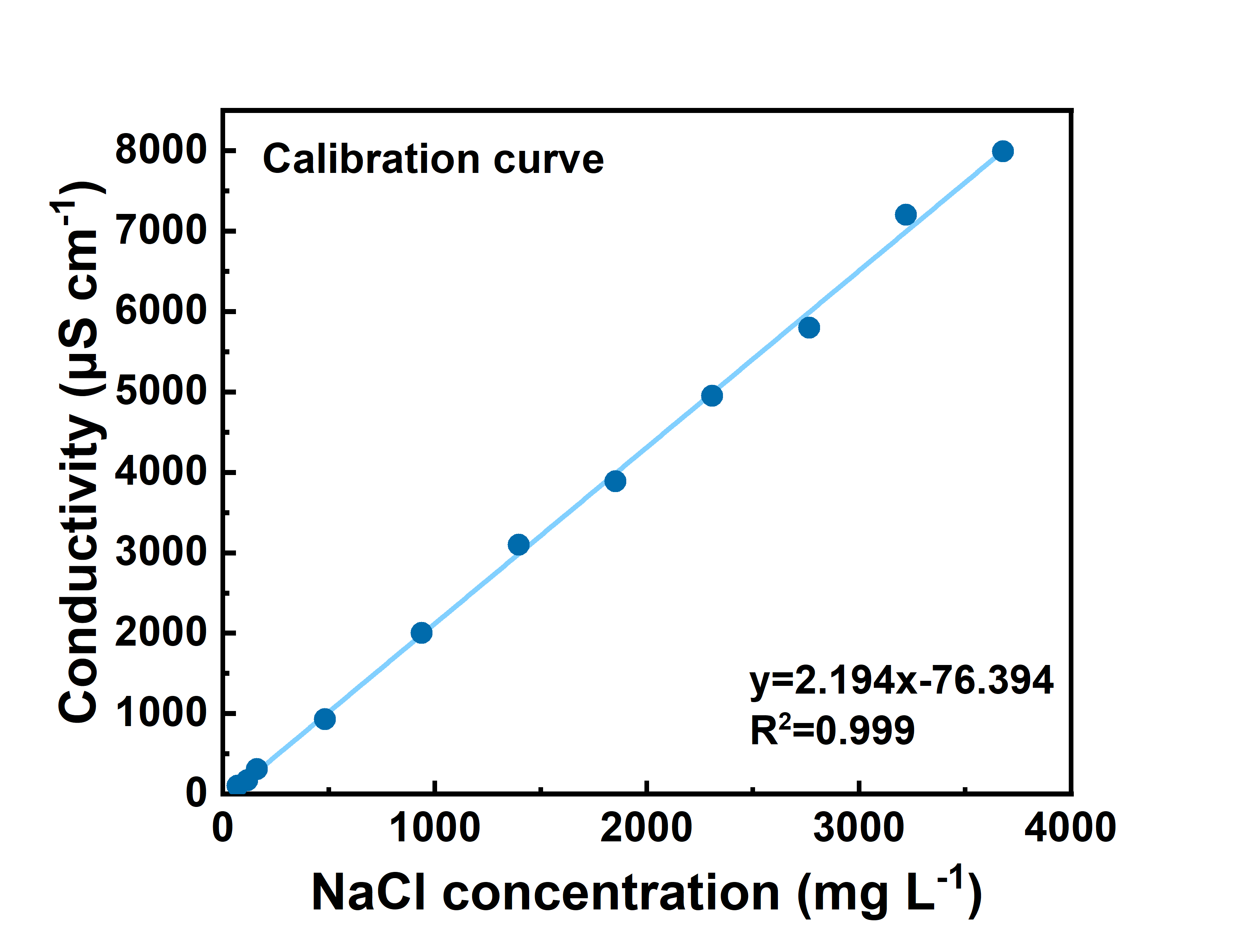


**Fig. S3** Calibration curve in conductivity as a function of NaCl concentration measured at room temperature (25 °C)


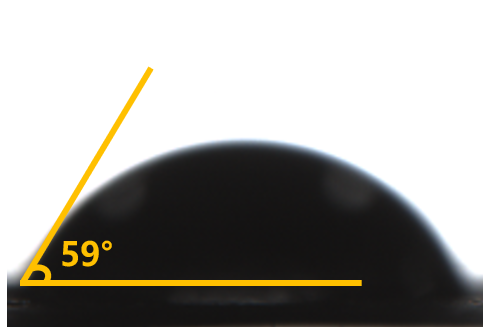


**Fig. S4** The static contact angle of the stainless-steel current collector


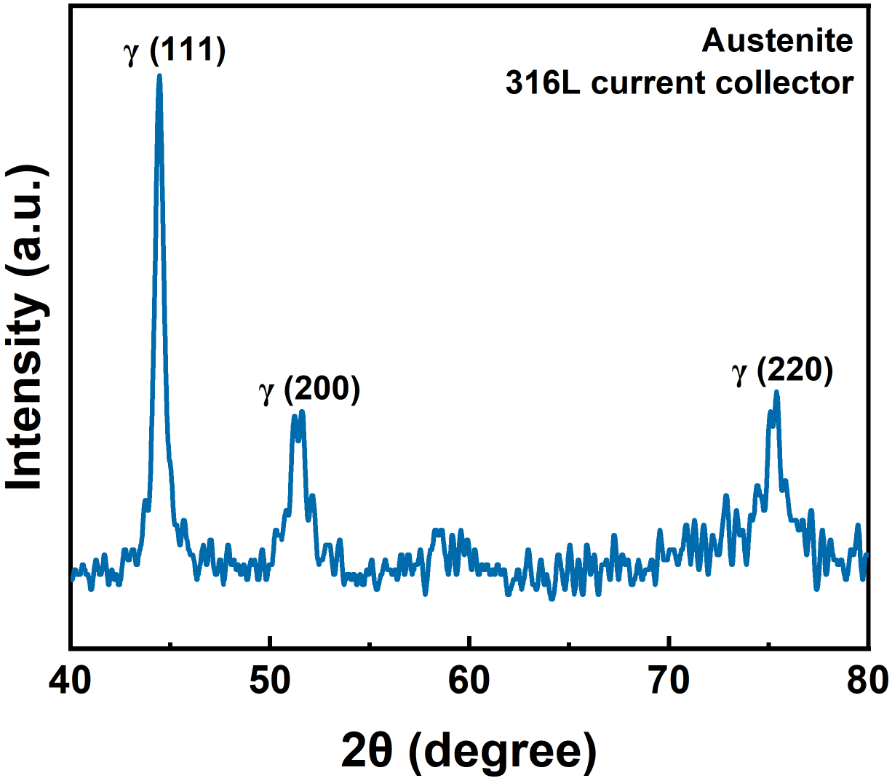


**Fig. S5** XRD pattern of the current collector made of 316L stainless steel


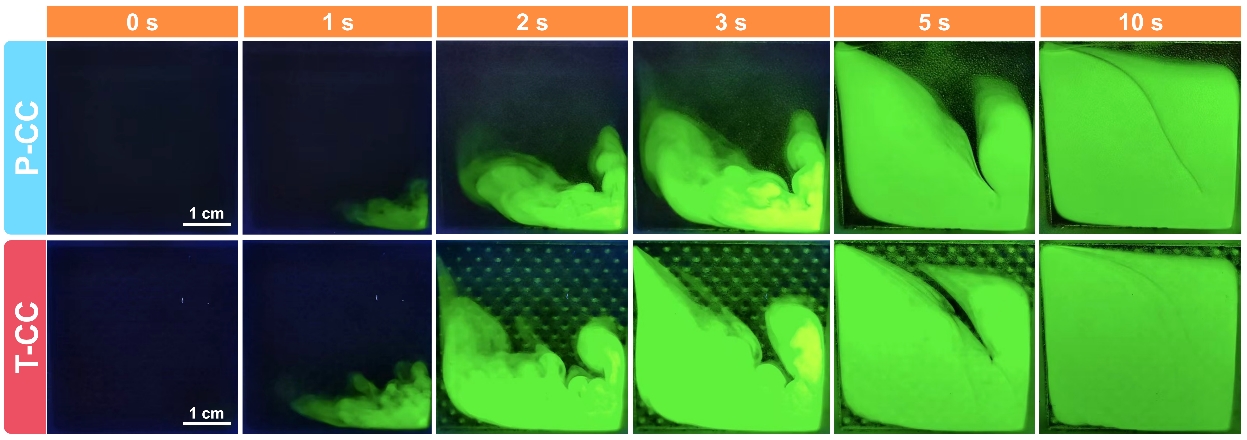


**Fig. S6** Digital images of the fluid transport and mixing in P-CC and T-CC at various time intervals


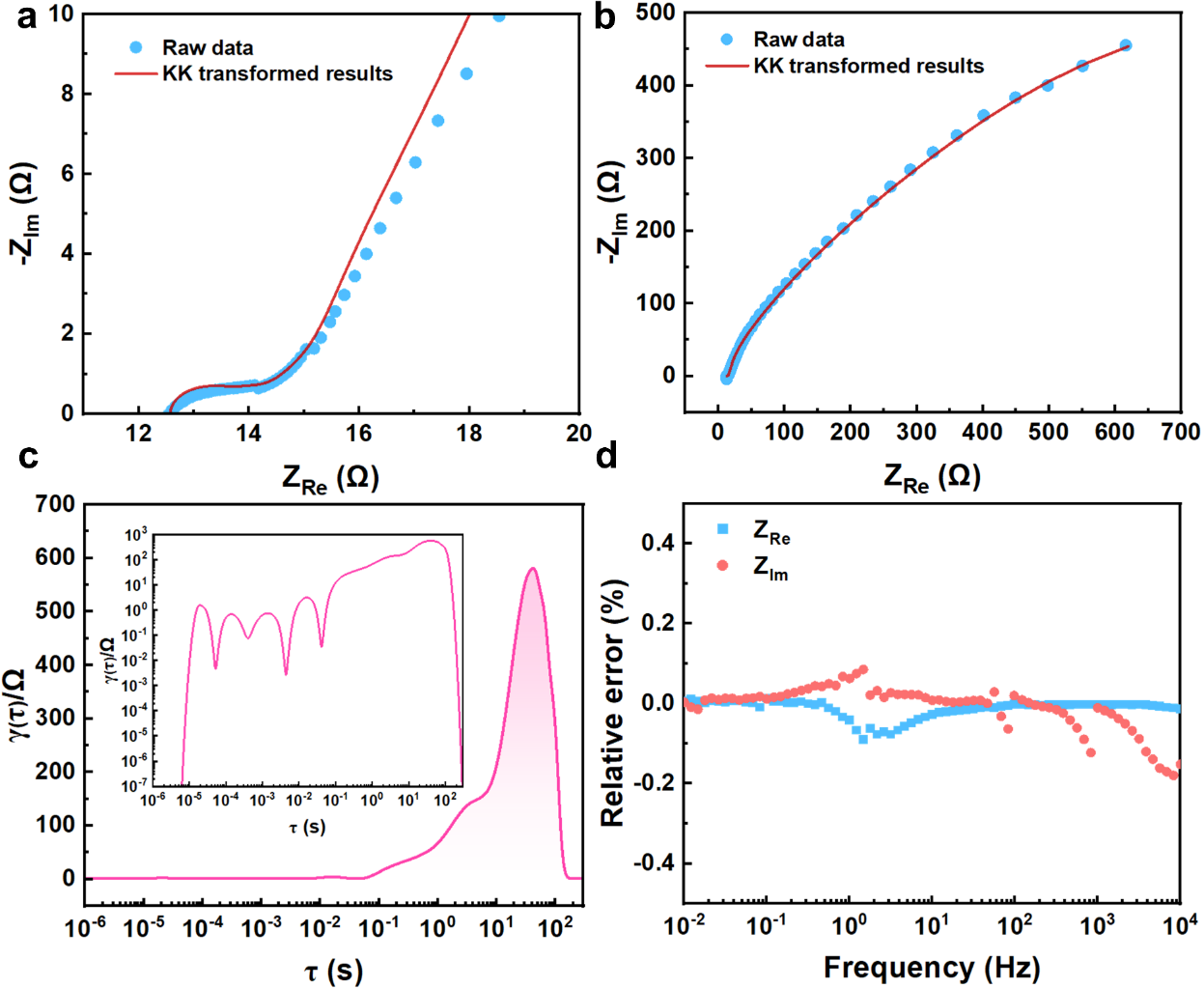


**Fig. S7** **(a)** High frequency region and **(b)** full frequency region of the Nyquist plot obtained from the FCDI system and its Kramers-Kronig transformed results; **(c)** DRT plots and **(d)** residual plots of the FCDI system


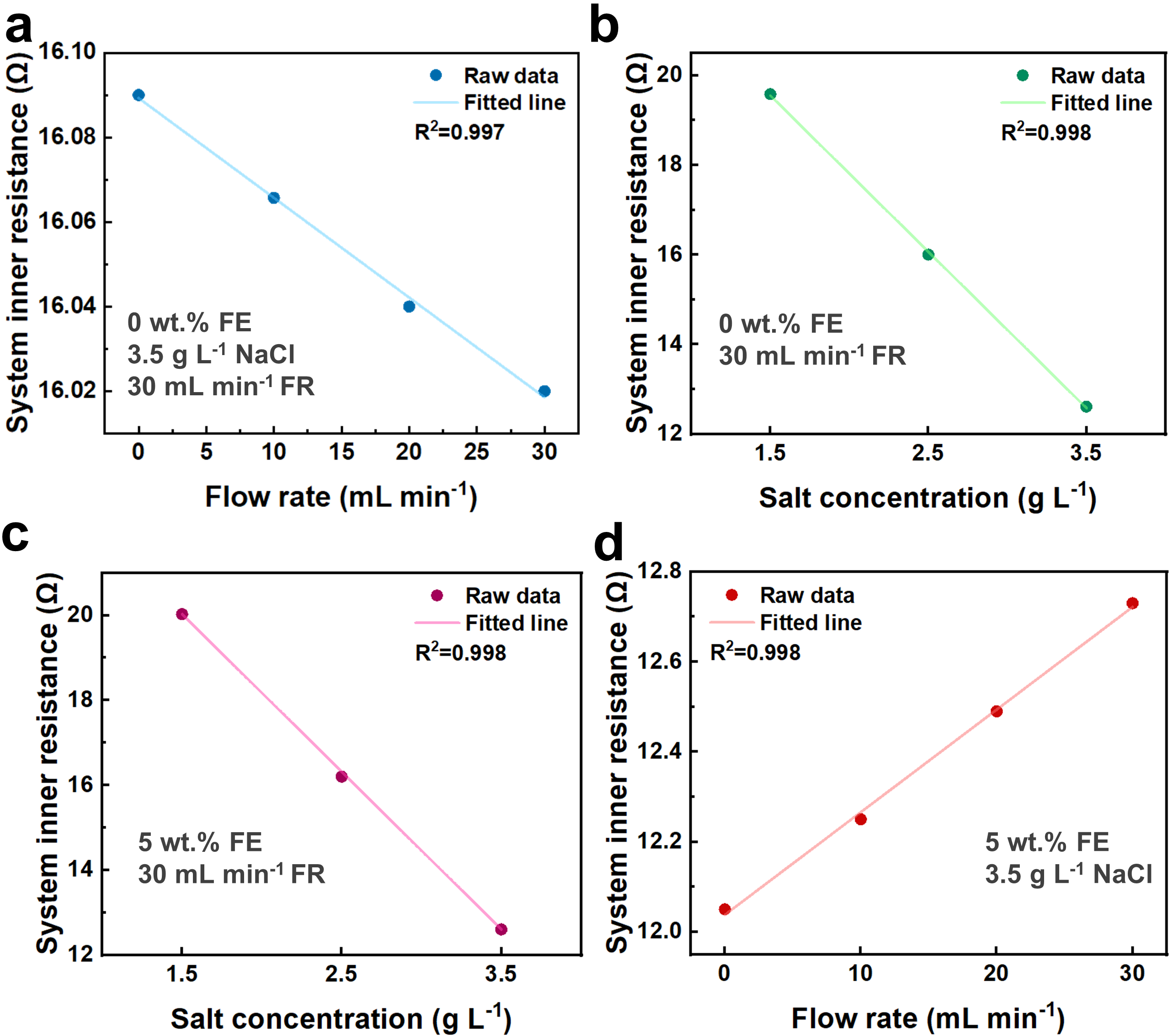


**Fig. S8** Dependence of the internal resistance of the ED system on: **(a)** flow rate, and **(b)** concentration of the saltwater; Dependence of the internal resistance of the FCDI systems on: **(c)** salt concentration, and **(d)** flow rate of the flow electrode


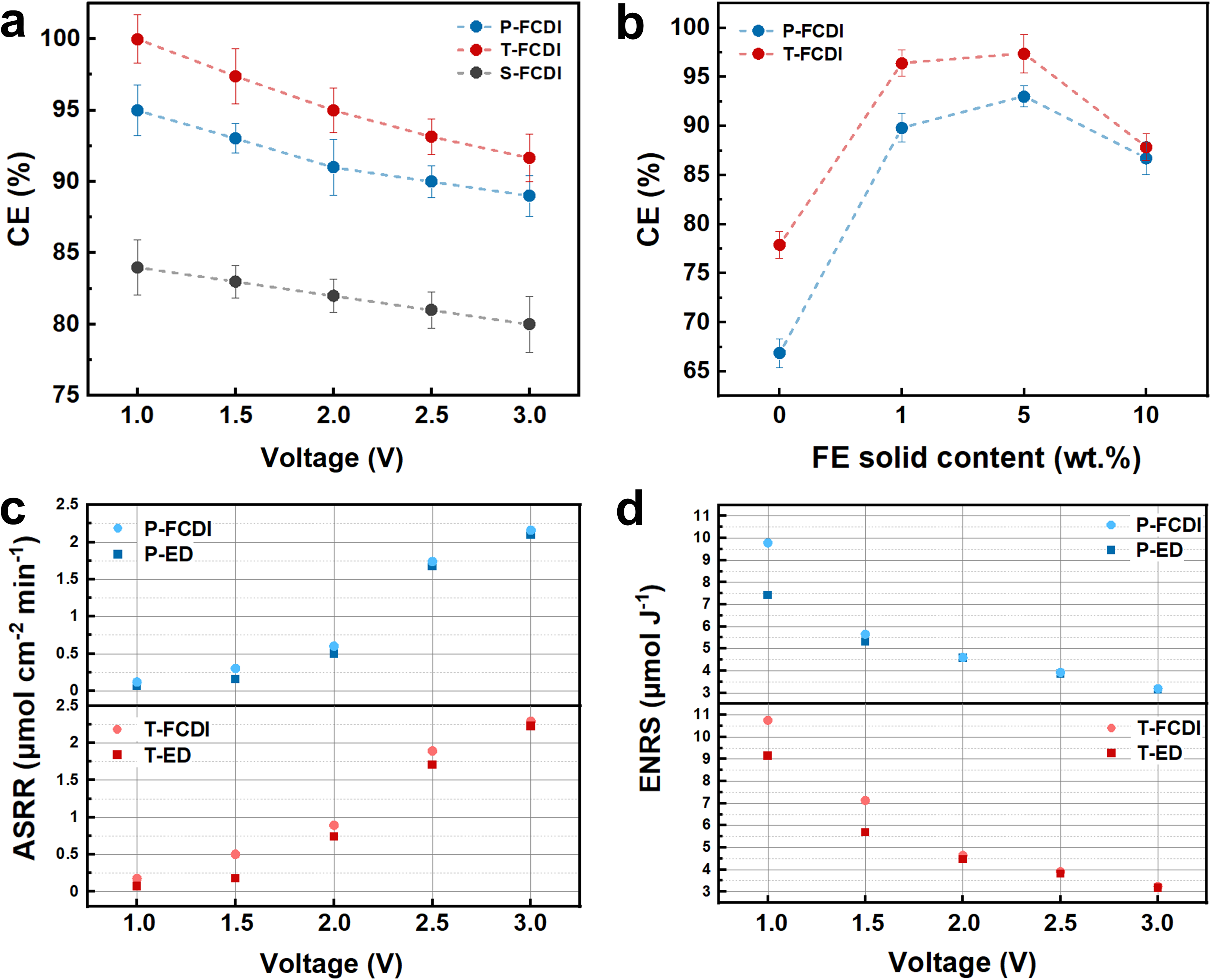


**Fig. S9 (a)** CE for S-FCDI, P-FCDI and T-FCDI; **(b)** CE for T-FCDI with different solid contents in the flow electrode (FE); **(c)** ASRR and **(d)** ENRS comparison plots of the ED and FCDI systems equipped with different current collectors


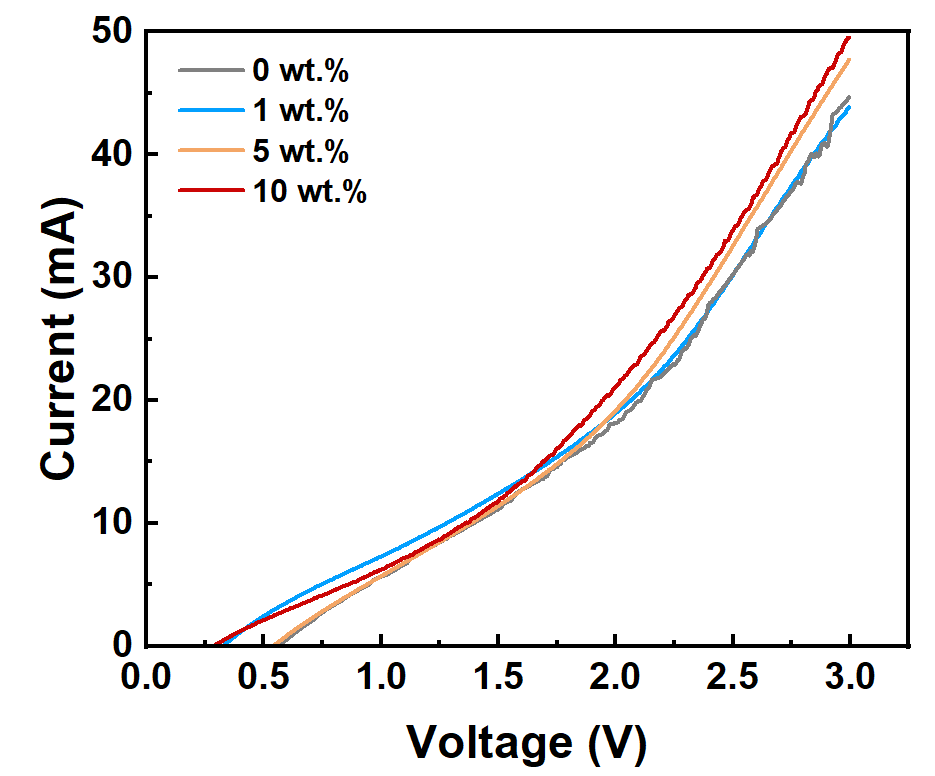


**Fig. S10** LSV curves of the T-T FCDI cells with different solid contents in the flow electrode


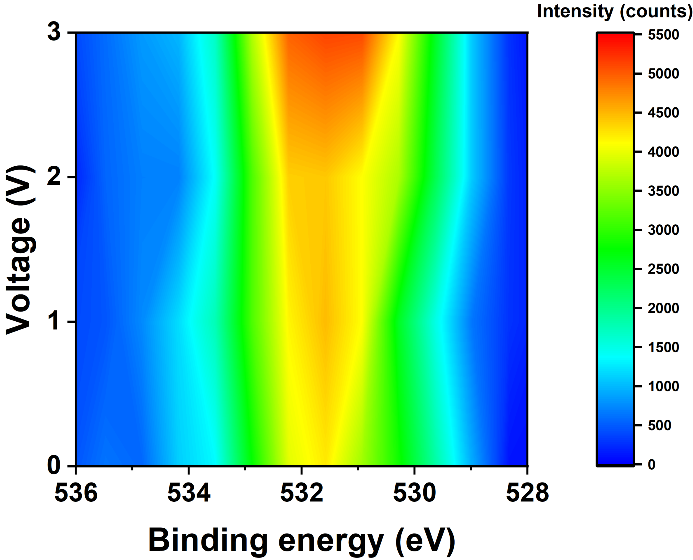


**Fig. S11** High-resolution XPS spectra in the O*1s* region of flow electrode (carbon) obtained after FCDI tests at different voltages


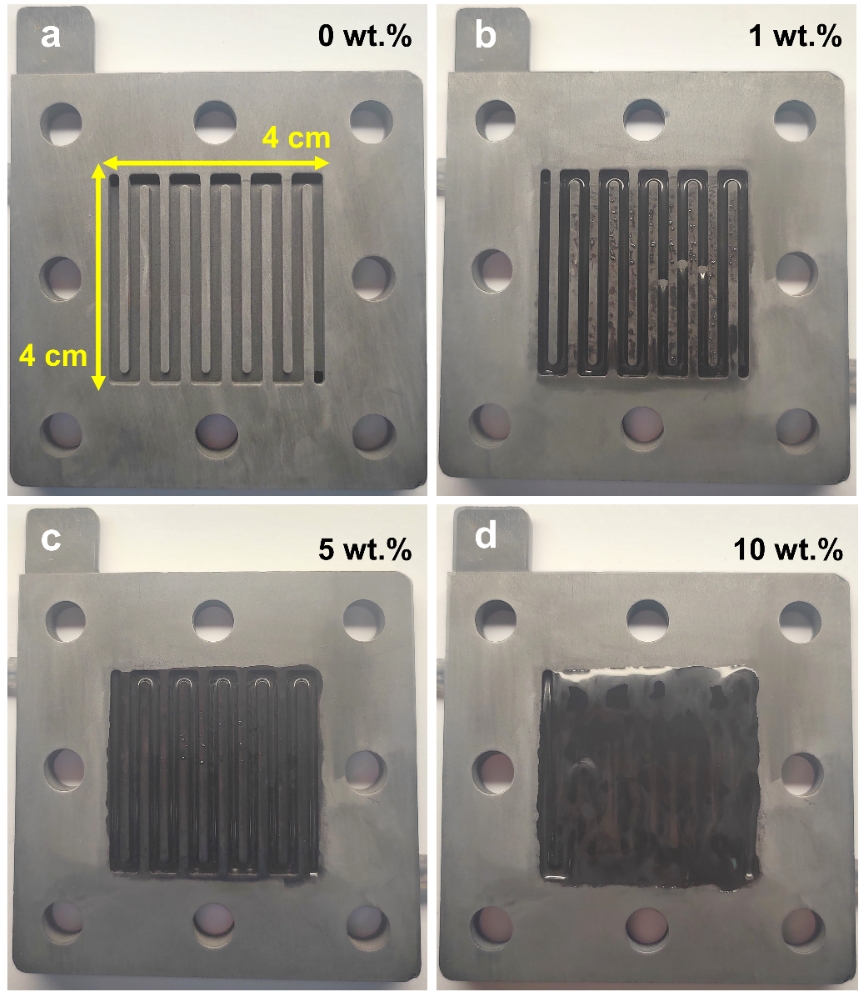


**Fig. S12** Digital images of the 2D current collector (S-CC) after FCDI tests, with a flow electrode solid content of (**a**) 0 wt.%, (**b**) 1 wt.%, (**c**) 5 wt.%, and (**d**) 10 wt.%


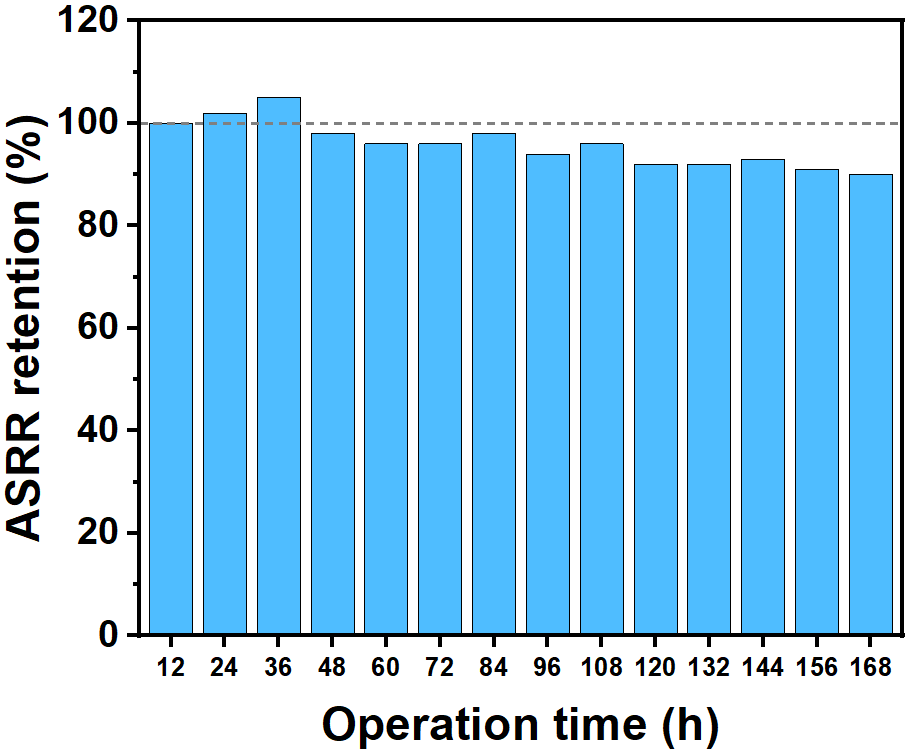


**Fig. S13** ASRR retention during 7 days of continuous operation

# S2 Supplementary Tables

**Table S1** The properties of the activated carbon (YP-50F) used in this study

| **Characteristics** | **YP-50F** |
| --- | --- |
| Surface area (m^2^ g^-1^) | 1692 |
| Bulk density (g mL^-1^) | 0.30 |
| Moisture (%) | 0.5 |
| Ash (%) | 0.2 |
| pH | 8.4 |
| Metal content (Fe) (ppm) | 11.0 |
| Size distribution (μm)  D10/D50/D90 | 1.9/5.6/9.7 |
| Weight capacitance (F g^-1^) | 28.6 |
| Volumetric capacitance (F cc^-1^) | 14.8 |

Note: Data were provided by the manufacturer (Kuraray Co., Ltd., Japan).

**Table S2** The properties of the ion exchange membranes used in this study

| **Characteristics** | **AEM** | **CEM** |
| --- | --- | --- |
| Grade | AMX | CMX |
| Type | Strongly basic | Strongly acidic |
| Thickness (mm) | 0.14 | 0.17 |
| Electrical resistance (Ω·cm^2^) | 2.4 | 3.0 |
| Burst strength (MPa) | ≥0.25 | ≥0.40 |

Note: Data were provided by the manufacturer (ASTOM Co., Ltd., Japan).

**Table S3** Geometric parameters of three kinds of current collectors

| Current collector type | Contact area (cm^2^) | Surface area (cm^2^) | Inner volume  (mL) |
| --- | --- | --- | --- |
| T-CC | 16 | 20.5 | 3.10 |
| P-CC | 16 | 19.2 | 3.20 |
| S-CC | 16 | 26.8 | 2.44 |

**Table S4** The properties of the current collectors fabricated in this study

| **Characteristics** | **316L stainless steel** |
| --- | --- |
| Chemical composition (wt.%) | 16~18% Cr, 10~14% Ni, 2~3% Mo, <2% Mn, <1% Si, <0.03% C, <1% Si, <0.045% P, <0.03% S, <0.13% O, and Fe balance |
| Density (g cm^-3^) | 8.03 |
| Electrical resistance (μΩ·cm) | 74 |
| Specific heat capacity (J kg^-1^ K^-1^) | 502 |
| Thermal conductivity (W m^-1^ K^-1^) | 16.3 |
| Yield strength (MPa) | > 170 |
| Tensile strength (MPa) | > 485 |
| Poisson's ratio | 0.28 |
| Static contact angle (°) | 59 |
| Roughness average | 7 |
| Porosity | < 1 |

**Table S5** Average flow velocities and maximum flow velocities data in Fig. 2d

| Inlet flow rate (mL min^-1^) | P-CC  Average flow velocity  (mm s^-1^) | T-CC  Average flow velocity  (mm s^-1^) | P-CC  Maximum flow velocity  (mm s^-1^) | T-CC  Maximum flow velocity  (mm s^-1^) |
| --- | --- | --- | --- | --- |
| 5 | 0.78 | 0.80 (2.56%) | 1.60 | 2.93 (82.73%) |
| 10 | 1.57 | 1.61 (2.57%) | 3.20 | 5.87 (83.29%) |
| 15 | 2.35 | 2.41 (2.57%) | 4.79 | 8.81 (83.95%) |
| 20 | 3.14 | 3.22 (2.54%) | 6.37 | 11.76 (84.78%) |
| 25 | 3.92 | 4.02 (2.49%) | 7.93 | 14.71 (85.48%) |
| 30 | 4.71 | 4.82 (2.42%) | 9.50 | 17.65 (85.90%) |

Note: The numbers in parentheses are the enhancement ratios.

**Table S6** Comparison of the performance with the literature data

| **Ref.** | **Operation mode** | **Initial salt concentration**  **(g L^-1^)** | **Voltage (V)** | **Solid content (wt.%)** | **ASRR**  **(μmol cm^-2^ min^-1^)** | **ENRS**  **(μmol J^-1^)** |
| --- | --- | --- | --- | --- | --- | --- |
| [S1] | SCC batch mode | 2.00 | 1.2 | 5.00 | 1.03 | 0.87 |
| [S2] | ICC batch mode | 15.00 | 1.2 | 5.00 | 0.05 | 3.38 |
| [S3] | SCC batch mode | 0.50 | 1.0 | 2.50 + 1.25 | 0.24 | 10.10 |
| [S4] | SCC batch mode | 2.15 | 0.8 | 2.00 + 0.20 | 0.08 | 12.20 |
| [S4] | SCC batch mode | 2.15 | 1.2 | 2.00 + 0.20 | 0.16 | 8.30 |
| [S5] | SCC batch mode | 3.50 | 1.2 | 12.50 | 0.25 | 5.73 |
| [S5] | SCC batch mode | Yellow sea water | 1.2 | 12.50 | 0.22 | 10.30 |
| [S5] | SCC batch mode | South China sea water | 1.2 | 12.50 | 0.40 | 9.60 |
| [S6] | ICC single pass | 0.50 | 1.2 | 0.10 | 0.11 | 6.59 |
| [S6] | ICC single pass | 0.50 | 1.2 | 0.50 | 0.15 | 6.98 |
| [S6] | ICC single pass | 0.50 | 1.2 | 2.50 | 0.19 | 7.09 |
| [S6] | ICC single pass | 0.50 | 1.2 | 5.00 | 0.21 | 7.54 |
| [S6] | ICC single pass | 0.50 | 1.2 | 10.00 | 0.23 | 7.78 |
| S-FCDI | SCC batch mode | 3.50 | 1.0 | 5.00 | 0.10 | 4.38 |
|  | SCC batch mode | 3.50 | 1.5 | 5.00 | 0.21 | 3.79 |
|  | SCC batch mode | 3.50 | 2.0 | 5.00 | 0.36 | 3.34 |
| P-FCDI | SCC batch mode | 3.50 | 1.0 | 5.00 | 0.12 | 9.79 |
|  | SCC batch mode | 3.50 | 1.5 | 5.00 | 0.30 | 5.65 |
|  | SCC batch mode | 3.50 | 2.0 | 5.00 | 0.59 | 4.60 |
| T-FCDI | SCC batch mode | 3.50 | 1.0 | 5.00 | 0.18 | 10.76 |
|  | SCC batch mode | 3.50 | 1.5 | 5.00 | 0.50 | 7.13 |
|  | SCC batch mode | 3.50 | 2.0 | 5.00 | 0.89 | 4.65 |

# Supplementary Notes

## S3.1 Kramers-Kronig (KK) validation and DRT analysis

Kramers-Kronig (KK) transformations were performed to validate the obtained EIS data, which ensure the investigated electrochemical system is stable and linear [S7]. The imaginary part of the impedance (*Z*_j,KK_(*ω*)) can be calculated from the real part (*Z*_r,KK_(*ω*)) and *vice versa* [S8]:

$$\begin{aligned} Z_{j,KK}\left( \omega\right)=\frac{2\omega}{\pi}\int_{0}^{\infty} \frac{Z_{j}\left( x \right)-Z_{j}\left( \omega\right)}{x^{2}-\omega^{2}}dx\#\left( S1 \right) \end{aligned}$$

$$\begin{aligned} Z_{r,KK}\left( \omega\right)=R_{\infty}+\frac{2}{\pi}\int_{0}^{\infty} \frac{xZ_{j}\left( x \right)-\omega Z_{j}\left( \omega\right)}{x^{2}-\omega^{2}}dx\#\left( S2 \right) \end{aligned}$$

where *ω* is the angular frequency, *x* is the angular frequency of any specific data point. The residuals between the measured data (*Z*_j_(*ω*) and *Z*_r_(*ω*)) and the KK transformation results (*Z*_j,KK_(*ω*) and *Z*_r,KK_(*ω*)) were calculated as follows:

$$\begin{aligned} \Delta Z_{j}\left( \omega\right)=\frac{\left( Z_{j}\left( \omega\right)-Z_{j,KK}\left( \omega\right) \right)}{\left| Z_{KK}\left( \omega\right) \right|}\#\left( S3 \right) \end{aligned}$$

$$\begin{aligned} \Delta Z_{j}\left( \omega\right)=\frac{\left( Z_{j}\left( \omega\right)-Z_{j,KK}\left( \omega\right) \right)}{\left| Z_{KK}\left( \omega\right) \right|}\#\left( S4 \right) \end{aligned}$$

The KK-validated impedance data were then fitted by the following DRT model: [8]

$$\begin{aligned} Z\left( \omega\right)=R_{\infty}+R_{\mathrm{pol}}\int_{0}^{\infty} \frac{\gamma\left( \tau\right)}{1+j\omega\tau}d\tau\#\left( S5 \right) \end{aligned}$$

where *Z*(*ω*) is the KK-validated impedance data obtained from the EIS measurements, R_∞_ is the ohmic resistance, R_pol_ is the overall polarization resistance of the real part of the impedance and *j* is the complex unit. The function *γ*(τ) is the normalized distribution of the relaxation time.

## S3.2 Computational methods

COMSOL^®^ Software Version 6.1 was used for all simulations in this work [S9].

### S3.2.1 CFD simulation model and flow particle tracking

A hydrodynamic simulation model was built to simulate the flow field distribution of the flow electrode in different current collectors. It was assumed that the density, conductivity and viscosity of the solution remain unchanged, and the flow electrode was isotropic, so it could be regarded as the simple Newtonian fluid (*i.e.,* incompressible and fully developed flow). The left boundary of the geometric model was set as the fluid inlet and the right boundary was set as the outlet. The flow rate was varied from 0 to 30 mL min^-1^. The fluid followed the no-slip condition. The flow state of the fluid was controlled by the Navier-Stokes equation of the laminar flow (Eq. S6) [S10] . Combined with the mass conservation equation (Eq. S7), the steady-state value of the flow electrode velocity could be solved.

$$\begin{aligned} \rho\left( u\cdot\nabla\right)u=\nabla\cdot\left[ -pl+K \right]+F+\rho g\#\left( S6 \right) \end{aligned}$$

$$\begin{aligned} \rho\nabla\cdot u=0\#\left( S7 \right) \end{aligned}$$

where u, ρ, p and v are the flow rate, density, pressure and kinematic viscosity of the fluid respectively.

Based on the CFD simulation results, the movements of the carbon particles in the flow channels were further simulated via the flow particle tracking module in the COMSOL software. It was estimated that 1 cm^3^ of the flow electrode with a solid content of 5 wt.% contained about 3.2×10^8^ carbon particles [S11]. Simulating such a large number of particles is extremely compute-intensive and unnecessary. Herein, the concept of "Super particle" (SP) [S12] was used to represent many weakly interacting real particles in a specific region. The building blocks of the model were not individual carbon particles, but groups of carbon particles. These SPs could be visualized as a small patch of space, each following the same path as a real carbon particle. The motion path of each SP was tracked based on Newton's laws of motion (Eq. S8) to simulate the approximate flow path of carbon particles in the slurry.

$$\begin{aligned} \frac{d\left( m_{p}v \right)}{dt}=\boldsymbol{F}t\#\left( S8 \right) \end{aligned}$$

where the m_p_ and *v* are the mass and the velocity of the super particle, *t* is the time, ***F*** is the force acting on the particle.

The left channel entrance was set as the particle release source, the release time was evenly distributed, and the number of released particles was adjusted with the change of the flow electrode solid content. The trajectory of any particle was solved in the time domain. At each time step set by the solver, the force acting on each particle was queried at its current position. The positions of all particles are given by *q* (*q*_x_, *q*_y_, *q*_z_), and their respective velocities at specific locations are given by *v* (*v*_x_, *v*_y_, *v*_z_).

### S3.2.2 Electric field model

A 3D electric field simulation model (the model size was the same as the real FCDI cell) was built based on the electrostatic field module in the COMSOL software to simulate the electric field and surface charge density distribution of the flow channels.

In this model, the electrostatic field distribution is defined according to the Faraday's Law (Eq. S9):

$$\begin{aligned} E=-\nabla V\#\left( S9 \right) \end{aligned}$$

where *E* is the electric field intensity, ∇*V* is the potential gradient.

The conductivity of 316L stainless steel electrode material used in this experiment was 1.4 MS m^-1^. Gauss's law (Eq. S10) was used to describe the electric field:

$$\begin{aligned} \nabla\cdot\left( \varepsilon_{0}\varepsilon_{r}\boldsymbol{E} \right)=\rho_{v}\#\left( S10 \right) \end{aligned}$$

where ε_0_ is the vacuum dielectric constant, ε_r_ is the dielectric function of the material, the dielectric constant of the electrolyte is set as 81, and the dielectric constant of 316L stainless steel as a theoretical conductor is ∞. We set the FCDI anode current collector to a constant potential of 0 V and the cathode current collector to a constant potential varied from 0 to 3.0 V. The electric field intensity and the surface charge density distribution of the current collectors were solved by combining the CFD simulation results.

## S3.3 Charge efficiency and three kinds of charge contributions

As shown in Fig. S14a, the total charge flow through the FCDI system is denoted as Q_Total_, which can be recorded by the electrochemical workstation. Q_Total_ can be divided into three parts: the ion transport charge (Q_I_), the faradaic reaction charge (Q_F_) and the dissipation charge (Q_D_) as we mentioned in the manuscript. Following this thought, we proposed an equivalent circuit model (as shown in Fig. S14b) of the FCDI system.


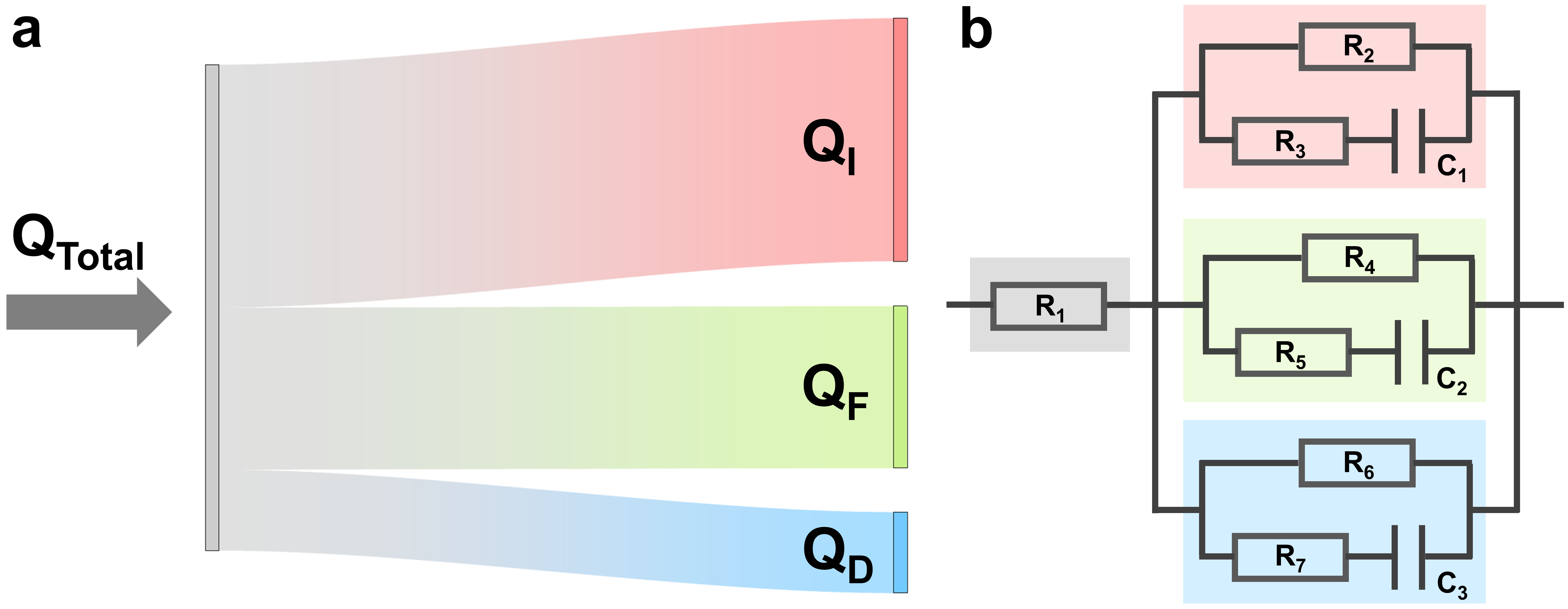


## Fig. S14 (a) charge flow diagram and (b) equivalent circuit model of the FCDI system

The three parallel circuits represent the ion transport process (red), faradaic reaction process (green), and the charge dissipation process (blue). The s ymbol R refers to the ideal resistance, and the symbol C refers to the ideal capacitor.

**R_1_** represents the resistance of the electron transfer process in the system (*i.e.,* the resistance of the electrical conductors). For example, it includes the resistance of the current collectors, the contact resistance between current collectors and particles, the contact resistance between carbon particles and the conductive carbon black, and the resistance of carbon particles and conductive carbon black themselves.

The ion transport process in FCDI can be represented by the equivalent circuit in the red box. All charges flowing through this branch are used for ion transport, which can be expressed by the Nernst–Planck (NP) equation [S13].Ion transport needs to overcome the transport resistance, which can be represented by **R_2_**. For example, it includes the transmembrane transport resistance, the transport resistance in the electrolyte, and the ion transfer resistance in the carbon pores. At the same time, the ion transport process also involves the formation of an imaginary electrical double layer (**C_1_**). According to the modified Donnan (mD) model [S14], the Stern layer voltage is the driving force for the capacitive ion adsorption, and this part participates in ion transport as well. Obviously, the formation of the double layer is not an ideal process, and it needs to be connected in series with a resistor (**R_3_**) to represent the resistance to the formation of the double layer.

The Faradaic reaction process in the FCDI cell can be represented by the equivalent circuit in the green box. For all electrochemical reactions that occur in the FCDI cell (such as water splitting, chlorine evolution, and carbon oxidation), they can be all abstracted as a single-electron oxidation-reduction reaction occurring at an imaginary electrode/electrolyte interface (defined as the reaction plane). This reaction needs to overcome the electron transfer barrier (**R_4_**). In the meanwhile, an imaginary electrical double layer forms at this imaginary interface, which is denoted as **C_2_**. The barrier of the electrical double layer forming process is denoted as **R_5_**, which is in series with **C_2_**, making the EDL capacitor nonideal. According to the generalized Frumkin–Butler–Volmer (gFBV) equation [S15], the smaller the electron transfer resistance is, the greater the exchange current density of the reaction is, and more easily the reaction occurs.

The charge dissipation process in the FCDI cell can be represented by the equivalent circuit in the blue box. The charge dissipation process mainly refers to the process where the electrons on carbon particles that are not involved in ion adsorption but neutralize with the charged particles flowing out from the other pole. At the same time, when the carbon particles come into contact with the salt solution or air, a small amount of charge neutralization occurs. Such charge dissipation processes need to overcome the interface charge transfer resistance **R_6_** (*e.g.,* the contact resistance between carbon particles). At the same time, the non-ideal double layer formed at this imaginary interface can also be represented by an RC series circuit (**R_7_** and **C_3_**).

**Supplementary References**

1. L. Yan, S. Issaka Alhassan, H. Gang, et al. Enhancing charge transfer utilizing ternary composite slurry for high-efficient flow-electrode capacitive deionization. Chem. Eng. J. **468**, 143413 (2023). <https://doi.org/https://doi.org/10.1016/j.cej.2023.143413>
2. Y. Gendel, A. K. E. Rommerskirchen, O. David, et al. Batch mode and continuous desalination of water using flowing carbon deionization (FCDI) technology. Electrochem. Commun. **46**, 152-156 (2014). <https://doi.org/10.1016/j.elecom.2014.06.004>
3. L. Q. Xu, S. Peng, K. Wu, et al. Precise manipulation of the charge percolation networks of flow-electrode capacitive deionization using a pulsed magnetic field. Water Res. **222**, 118963 (2022). <https://doi.org/10.1016/j.watres.2022.118963>
4. C. He, J. Ma, C. Zhang, et al. Short-circuited closed-cycle operation of flow-electrode CDI for brackish water softening. Environ. Sci. Technol. **52**, 9350-9360 (2018). <https://doi.org/10.1021/acs.est.8b02807>
5. X. Y. Zhang, H. J. Zhou, Z. He, et al. Flow-electrode capacitive deionization utilizing three-dimensional foam current collector for real seawater desalination. Water Res. **220**, 118642 (2022). <https://doi.org/10.1016/j.watres.2022.118642>
6. L. Q. Xu, Y. F. Mao, Y. Zong, et al. Scale-up desalination: Membrane-current collector assembly in flow-electrode capacitive deionization system. Water Res. **190**, 116782 (2021). <https://doi.org/10.1016/j.watres.2020.116782>
7. S. Wang, J. Zhang, O. Gharbi, et al. Electrochemical impedance spectroscopy. Nat. Rev. Methods Primers. **1**, 41 (2021). <https://doi.org/10.1038/s43586-021-00039-w>
8. B. A. Boukamp. A linear Kronig‐Kramers transform test for immittance data validation. J. Electrochem. Soc. **142**, 1885 (1995). <https://doi.org/10.1149/1.2044210>
9. S. COMSOL AB, Sweden. COMSOL Multiphysics® v. 6.1. cn.comsol.com. (2023)
10. A. Carmona-Orbezo, R. A. W. Dryfe. Understanding the performance of flow-electrodes for capacitive deionization through hydrodynamic voltammetry. Chem. Eng. J. **406**, 126826 (2021). <https://doi.org/https://doi.org/10.1016/j.cej.2020.126826>
11. F. Summer, J. Torop, A. Aabloo, et al. Particle dynamics-based stochastic modeling of carbon particle charging in the flow capacitor systems. Appl. Sci. **12**(4), 1887 (2022). <https://doi.org/10.3390/app12041887>
12. T. Z. Esirkepov. Exact charge conservation scheme for Particle-in-Cell simulation with an arbitrary form-factor. Comput. Phys. Commun. **135**, 144-153 (2001). <https://doi.org/https://doi.org/10.1016/S0010-4655(00)00228-9>
13. X. He, W. Chen, F. Sun, et al. Enhanced NH_4_^+^ removal and recovery from wastewater using Na-Zeolite-based flow-electrode capacitive deionization: insight from ion transport flux. Environ. Sci. Technol. **57**, 8828-8838 (2023). <https://doi.org/10.1021/acs.est.3c02286>
14. S. Porada, R. Zhao, A. van der Wal, et al. Review on the science and technology of water desalination by capacitive deionization. Prog. Mater. Sci. **58**, 1388-1442 (2013). <https://doi.org/10.1016/j.pmatsci.2013.03.005>
15. P. M. Biesheuvel, Y. Q. Fu, M. Z. Bazant. Diffuse charge and Faradaic reactions in porous electrodes. Phys. Rev. E. **83**, 061507 (2011). <https://doi.org/10.1103/PhysRevE.83.061507>
